# Spatiotemporal and genetic regulation of A-to-I editing throughout human brain development

**DOI:** 10.1016/j.celrep.2022.111585

**Published:** 2022-11-01

**Authors:** Winston H. Cuddleston, Xuanjia Fan, Laura Sloofman, Lindsay Liang, Enrico Mossotto, Kendall Moore, Sarah Zipkowitz, Minghui Wang, Bin Zhang, Jiebiao Wang, Nenad Sestan, Bernie Devlin, Kathryn Roeder, Stephan J. Sanders, Joseph D. Buxbaum, Michael S. Breen

**Affiliations:** 1 Seaver Autism Center for Research and Treatment, Icahn School of Medicine at Mount Sinai, New York, NY 10029, USA; 2 Department of Psychiatry, Icahn School of Medicine at Mount Sinai, New York, NY 10029, USA; 3 Department of Genetics and Genomic Sciences, Icahn School of Medicine at Mount Sinai, New York, NY 10029, USA; 4 Pamela Sklar Division of Psychiatric Genomics, Icahn School of Medicine at Mount Sinai, New York, NY 10029, USA; 5 Department of Psychiatry and Behavioral Sciences and UCSF Weill Institute for Neurosciences, University of California, San Francisco, San Francisco, CA 94158, USA; 6 Mount Sinai Center for Transformative Disease Modeling, Icahn School of Medicine at Mount Sinai, One Gustave L. Levy Place, New York, NY 10029, USA; 7 Icahn Institute for Genomics, Icahn School of Medicine at Mount Sinai, One Gustave L. Levy Place, New York, NY 10029, USA; 8 Department of Biostatistics, University of Pittsburgh, 130 De Soto Street, Pittsburgh, PA 15261, USA; 9 Department of Neuroscience and Kavli Institute for Neuroscience, Yale School of Medicine, New Haven, CT 06510, USA; 10 Program in Cellular Neuroscience, Neurodegeneration, and Repair and Yale Child Study Center, Yale School of Medicine, New Haven, CT 06510, USA; 11 Department of Psychiatry, Yale University School of Medicine, New Haven, CT 06520, USA; 12 Department of Genetics, Yale University School of Medicine, New Haven, CT 06520, USA; 13 Department of Comparative Medicine, Program in Integrative Cell Signaling and Neurobiology of Metabolism, Yale School of Medicine, New Haven, CT 06510, USA; 14 Department of Psychiatry, University of Pittsburgh School of Medicine, 3811 O’Hara Street, Pittsburgh, PA 15213, USA; 15 Carnegie Mellon University, Statistics & Data Science Department, Pittsburgh, PA 15213, USA; 16 Mindich Child Health and Development Institute, Icahn School of Medicine at Mount Sinai, New York, NY 10029, USA; 17 Friedman Brain Institute, Icahn School of Medicine at Mount Sinai, New York, NY 10029, USA; 18 These authors contributed equally; 19 Senior author; 20 Lead contact

## Abstract

Posttranscriptional RNA modifications by adenosine-to-inosine (A-to-I) editing are abundant in the brain, yet elucidating functional sites remains challenging. To bridge this gap, we investigate spatiotemporal and genetically regulated A-to-I editing sites across prenatal and postnatal stages of human brain development. More than 10,000 spatiotemporally regulated A-to-I sites were identified that occur predominately in 3′ UTRs and introns, as well as 37 sites that recode amino acids in protein coding regions with precise changes in editing levels across development. Hyper-edited transcripts are also enriched in the aging brain and stabilize RNA secondary structures. These features are conserved in murine and non-human primate models of neurodevelopment. Finally, thousands of *cis*-editing quantitative trait loci (edQTLs) were identified with unique regulatory effects during prenatal and postnatal development. Collectively, this work offers a resolved atlas linking spatiotemporal variation in editing levels to genetic regulatory effects throughout distinct stages of brain maturation.

## In brief

This resource article illuminates precise regulation of A-to-I editing throughout human brain development. These findings provide an atlas of spatiotemporally and genetically regulated A-to-I sites and their putative functional effects during prenatal and postnatal periods. Additional avenues to dissect the role of RNA editing in neurodevelopment are provided.

## Graphical Abstract

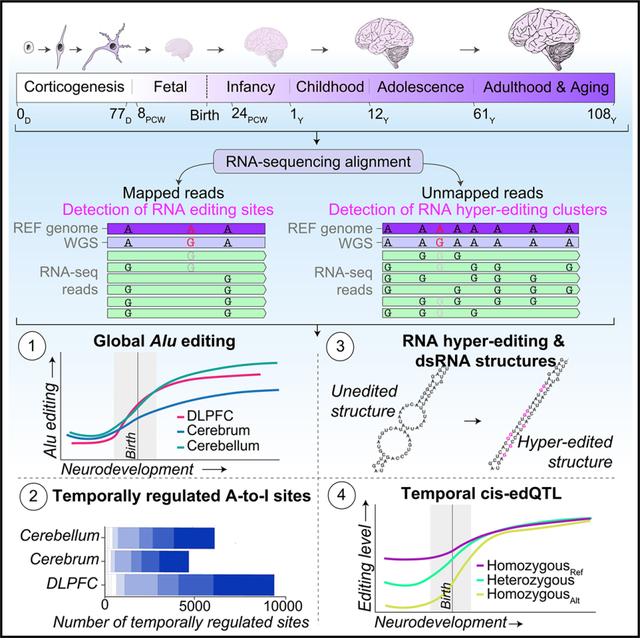

## INTRODUCTION

Adenosine-to-inosine (A-to-I) editing is a major contributor to the global diversity of RNA sequences in the human brain^1,2^ and is predicted to occur at over one hundred million locations in the human transcriptome.^3–5^ These base-specific modifications amplify the functionality of many neuronally expressed genes, including vital mediators of synaptic transmission and neuronal signaling.^1,2,6–9^ A-to-I editing in protein-coding regions can lead to amino acid substitutions. These “recoding” sites are required for typical neurodevelopment because they tightly regulate of Ca^2+^ permeability,^10,11^ enhance recovery rates from desensitization,^12^ and remodel actin cytoskeleton at excitatory synapses,^13,14^ among other functions.^15,16^ Importantly, many, if not most, physiologically crucial A-to-I sites illustrate a precise increase in editing levels throughout the various stages of neurodevelopment. Yet, as the catalog of A-to-I editing sites continues to expand, both the function and temporal profiles for most sites remain uncharted.

A-to-I editing occurs at single isolated adenosines (“selective editing”)^17,18^ or across many neighboring adenosines in an extended region (“hyper-editing”)^19–21^ and is catalyzed by family of adenosine deaminases acting on RNA (ADAR) enzymes. In mice, the spatiotemporal, cellular, and functional properties of essential A-to-I recoding sites in Gria2 (Q/R and R/G sites), *Grik1* and *Grik2* (Q/R), Gabra3 (I/M), and Cyfip2 (K/E), among others, have been extensively studied.^22–25^ These sites increase in editing levels across cortical developmental. Yet, the landscape of A-to-I editing is vastly under-represented in murine models since RNA editing is enriched in primate-specific *Alu* elements. In humans, our current knowledge base of temporally regulated A-to-I editing sites in the brain is subject to 742 selective editing sites that increase in editing levels across 9 fetal and 24 postnatal cortical samples, the majority in non-coding regions and show specific editing level changes in experimental models of brain disease.^26^ To build upon these efforts, a large-scale systematic approach to uncover spatiotemporally regulated A-to-I editing sites across large collections of deeply sequenced prenatal and postnatal tissues is warranted. Such an approach will be central for fully elucidating the landscape of temporally regulated A-to-I editing sites in the human brain.

Based on previous work, we reason that A-to-I sites that display discrete spatiotemporal differences in editing levels may signal a potentially critical role in supporting the functional diversity of brain development. Moreover, elucidating the genetic regulation of RNA editing at these sites may also offer unique insight into their role in both health and disease,^27–31^ and it has not yet been dissected across fetal and postnatal developmental periods.

The current study is anchored around *state-of-the-art* paired whole-genome and bulk-tissue RNA sequencing (RNA-seq) data from the dorsolateral prefrontal cortex (DLPFC) of 176 donors spanning 6 post-conception weeks (PCWs) to young adulthood (20 years).^32^ We cross-validated these findings in two independent developmental brain datasets of the cerebrum (n = 55) and cerebellum (n = 59).^33^ By combining these data with complementary RNA-seq data from *in vitro* models of corticogenesis (n = 151),^34,35^ hundreds of postmortem cortical tissues from advanced stages of normal aging (n = 261),^36^ and nonhuman primate (n = 96)^37^ and murine models of cortical development (n = 18),^38^ we provide comprehensive analysis and validation of the spatiotemporal regulation of A-to-I editing in the brain. Furthermore, we elucidate RNA editing quantitative trait loci (edQTLs), which act during prenatal and/or postnatal development. Collectively, this work provides a catalog of spatiotemporal and genetically regulated A-to-I editing sites throughout the widely different stages of human brain development.

## RESULTS

### Global editing levels increase across brain development and neuronal maturation

**Figure 1.**
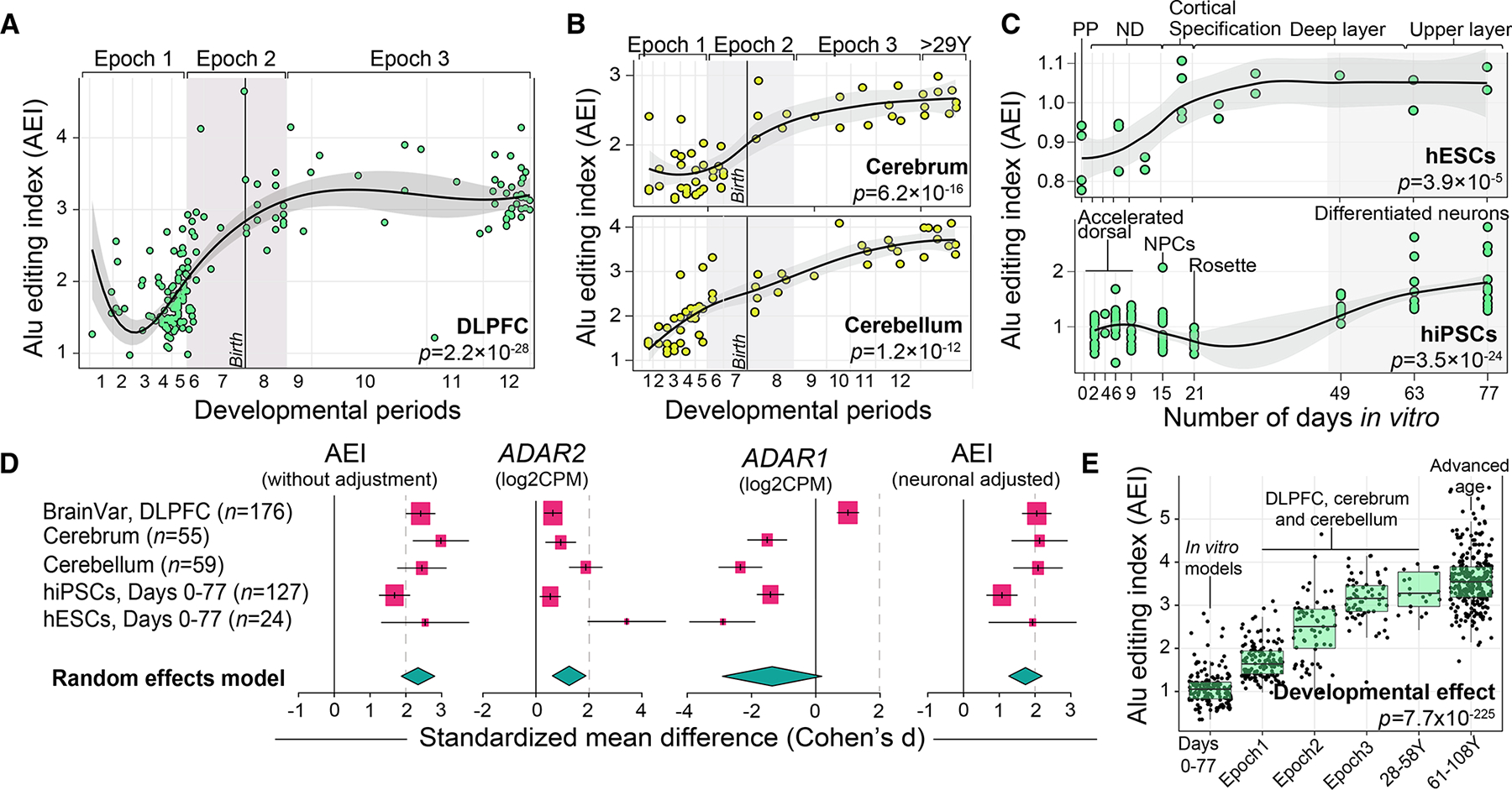
*Alu* editing index throughout human brain development and neuronal maturation (A and B) *Alu* editing index (AEI; y axis) was computed for (A) DLPFC (n = 176), (B) cerebrum (n = 55) and cerebellum (n = 59), across 12 developmental periods (log age, x axis). Periods 1–7 reflect prenatal windows and periods 8–12 reflect postnatal windows. The late fetal transitional period (epoch 2) is shaded in gray. (C) The AEI (y axis) throughout 77 days of neuronal maturation (x axis) in human embryonic stem cells (hESC; n = 24) and human induced pluripotent stem cells (hiPSCs; n = 127). Abbreviations depicting specific stages are described in the STAR Methods. Loess curves were used to fit the data. Two-sided linear regression was used to test for significance. (D) Meta-analysis of the AEI (with and without neuronal adjustment), *ADAR2*, and *ADAR1* across all datasets. Standardized mean difference (Cohen’s d) compared the differential change in these measures over the course of neuronal maturation and development. A random effects model computed the pooled effect size across all five independent datasets. Confidence intervals (95%) are denoted around each effect size and the size of each box scales with the relative sample size of each study. (E) The AEI (y axis) compiled across 702 developmentally distinct transcriptome samples (x axis), including samples from normal aging (n = 261). Two-sided linear regression was used to test for significance. All boxplots show the medians (horizontal lines), upper and lower quartiles (inner box edges), and 1.5× the inter-quartile range (whiskers).

A-to-I nucleoside modifications driven by ADAR enzymes are highly enriched in *Alu* elements. Thus, to quantify global selective editing levels we computed an *Alu* editing index (AEI)^39^ per donor, defined as the total number of A-to-G mismatches over the total coverage of adenosines in *Alu* elements (Table S1; Figure S1). We observe a 3-fold increase in global *Alu* editing across cortical development (p = 2.2 × 10^−28^, Cohen’s d = 2.55), with a major shift between the mid-fetal period and infancy (Figure 1A). To validate this temporal profile, the AEI was modeled across two smaller independent bulk RNA-seq datasets of the cerebrum (n = 55) and cerebellum (n = 59) spanning fetal and postnatal periods (4 PCWs to 58 years) and two experimentally tractable *in vitro* models of corticogenesis: (1) human embryonic stem cell (hESCs; n = 24), covering stages of pluripotency through upper layer generation (day 0–77); and (2) human induced pluripotent stem cells (hiPSCs) (n = 127), covering early differentiating cells through mature differentiated neurons (day 2–77). We observed a significant increase in the AEI across all datasets, including the cerebrum (p = 6.2 × 10^−16^, Cohen’s d = 2.98), cerebellum (p = 1.2 × 10^−12^, Cohen’s d = 2.45) (Figure 1B), hESCs (p = 3.9 × 10^−15^, Cohen’s d = 2.5), and hiPSC *in vitro* models (p = 3.5 × 10^−24^, Cohen’s d = 1.68) (Figure 1C). A meta-analysis synthesized these findings and validated an increase in global selective editing levels across neuronal maturation and brain development (pooled Cohen’s d = 2.34) (Figure 1D).

A matching meta-analytical framework confirmed a strong postnatal bias in *ADAR2* expression across all datasets (pooled Cohen’s d = 1.26) relative to a general prenatal bias for *ADAR1* (pooled Cohen’s d = −1.36) (Figure 1D), suggesting that the temporal *Alu* editing profiles may be *ADAR2* dependent. Given *ADAR2* is highly expressed in adult inhibitory and excitatory neurons,^31^ we estimated cellular proportions from bulk RNA-seq data and revealed expected postnatal bias in the proportions of mature neurons per donor (Figure S2). Following, we adjusted each of our analyses for the proportions of mature neurons per donor, and a significant postnatal bias in global *Alu* editing remained after this correction (pooled Cohen’s d = 1.84), suggesting that these changes are not fully driven by underlying cell-type composition (Figure 1D).

Given the substantial change in AEI between prenatal and postnatal periods, we next aimed to resolve any changes in *Alu* editing during advanced stages of aging. The AEI was computed for 261 additional cortical samples from older adults (61–108 years) (Figure S3). While editing levels were not dynamically regulated during these advanced stages of aging (p = 0.39) (Figure S3A), we compiled the AEI across all datasets and observed a steady increase in global *Alu* editing throughout all stages of neuronal maturation and brain development (p = 7.7 × 10^−225^) (Figure 1E), peaking between ~30 and 59 years of age. This association was also robust to neuronal cell-type correction (p = 4.2 × 10^−121^).

### Identification of high-confidence A-to-I editing sites across development

**Figure 2.**
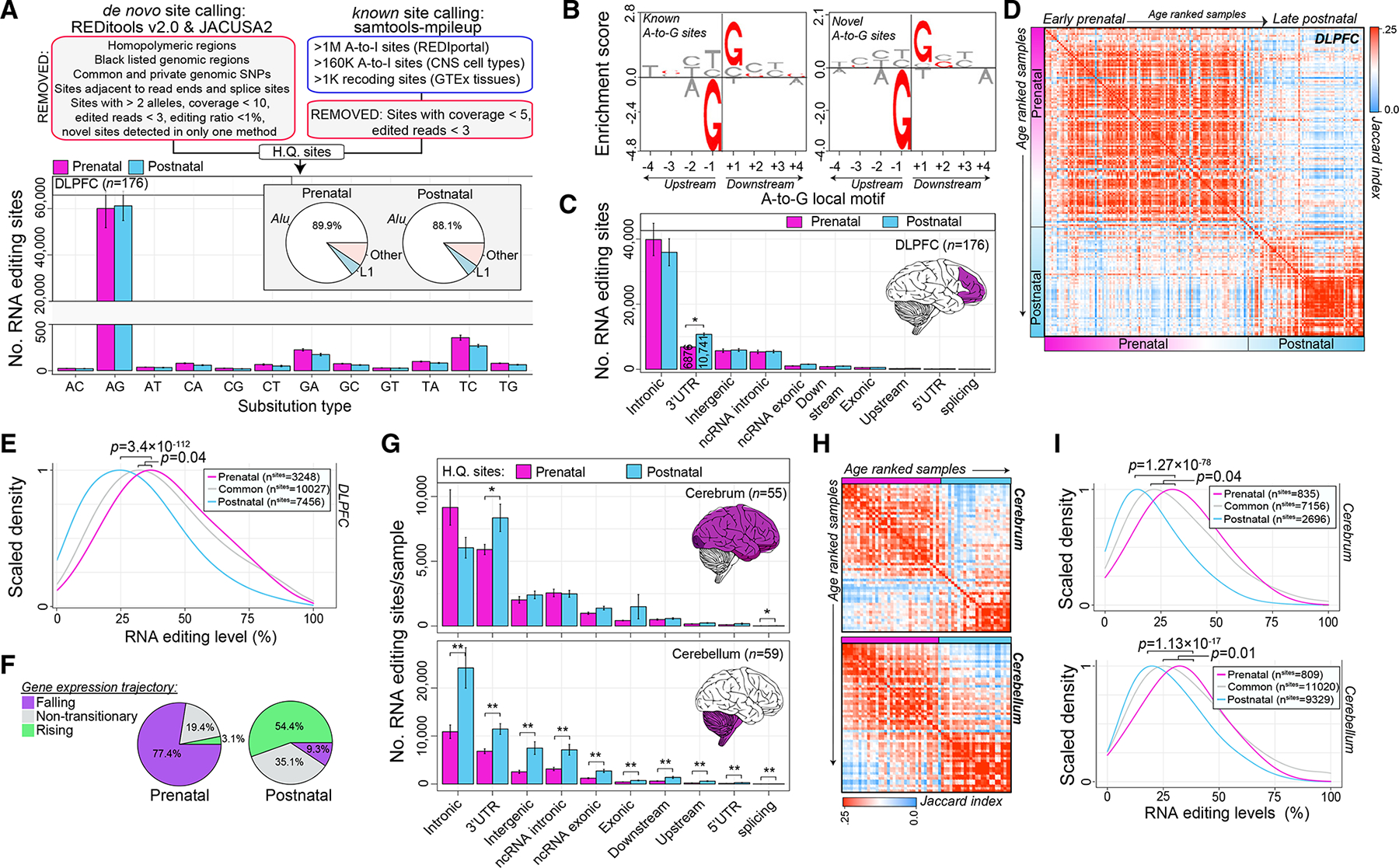
Identification and annotation of selective editing sites (A) Uncovering high-quality (HQ) sites (top). Bar plots depict mean (with standard error) number of HQ sites for DLPFC prenatal (n = 116) and postnatal (n = 60) samples based on substitution type and repeat element (bottom). (B) Known and not-in-catalog A-to-G sites enrich (y axis) for a common sequence motif featuring a depletion and enrichment of guanosines 1 bp (±) the target adenosine. (C) Bar plots depict mean number of sites (with standard error) per genic region for prenatal and postnatal samples, respectively. Two-sided Student’s t test tested for significance. (D) Jaccard index measures pairwise overlaps of HQ sites detected per sample (red, high; blue, low). Samples are age ranked from early fetal to late postnatal ages. (E) Differences in editing levels for prenatal specific sites relative to common and postnatal specific sites in the DLPFC. Two-sided Mann-Whitney U test tested for significance. (F) Prenatal- and postnatal-specific sites parsed by corresponding temporal gene expression trajectories. (G) Bar plots depicting mean (with standard error) number of HQ sites per genic region for cerebrum (n = 55; top) and cerebellum (n = 59; bottom) samples. Two-sided Student’s t test tested for significance. (H) Jaccard index measures pairwise overlaps of all HQ sites detected per sample in the cerebrum (top) and cerebellum (bottom). (I) Differences in editing levels for prenatal specific sites relative to common and postnatal specific sites in the cerebrum (top) and cerebellum (bottom). Two-sided Mann-Whitney U test tested for significance.

**Figure 3.**
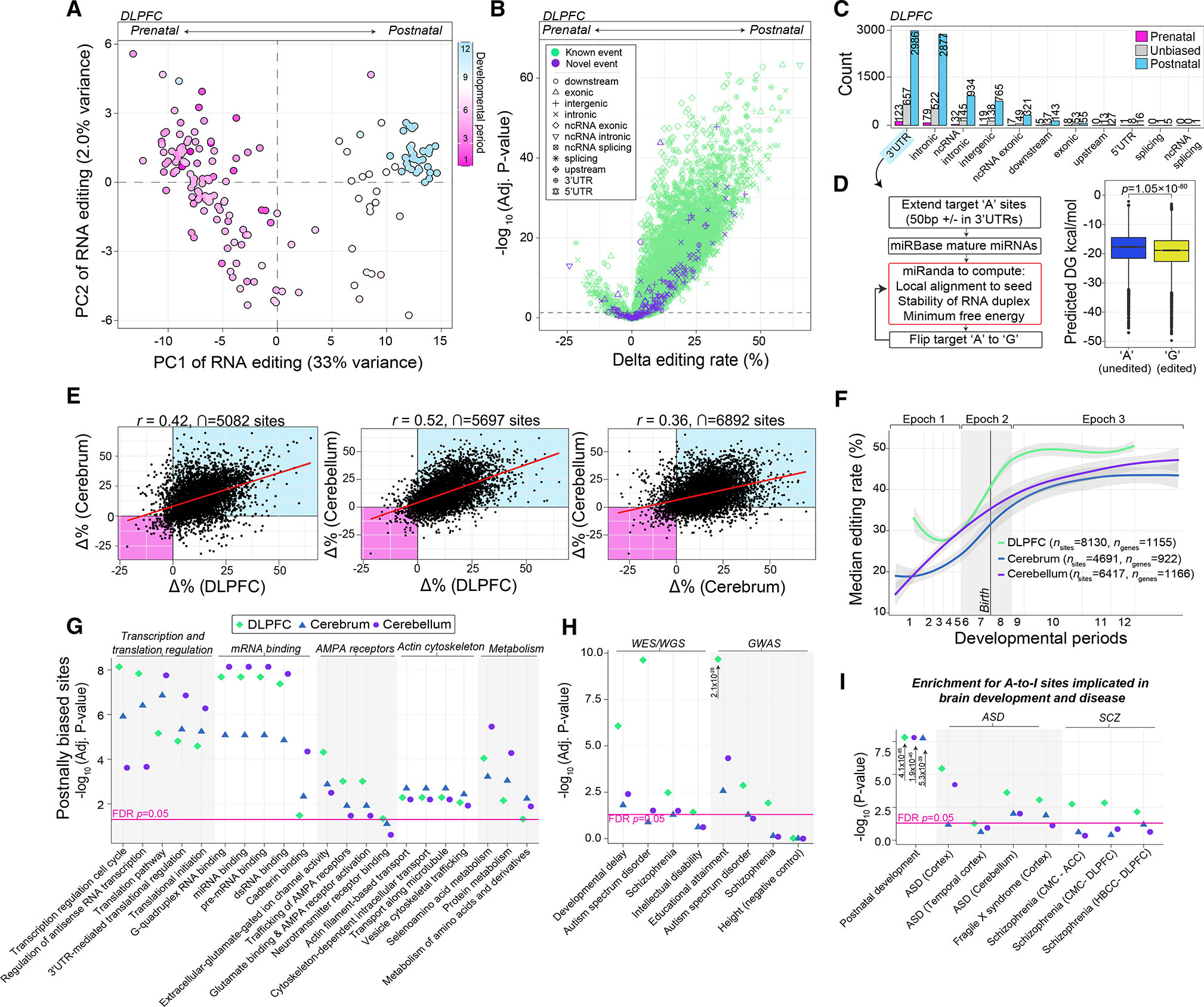
Spatiotemporal changes in RNA editing levels (A) Principal-component analysis of editing levels (n = 10,027 sites) stratifies DLPFC prenatal from postnatal samples (n = 176). (B) Differential editing analysis compares the strength of significance (−log_10_ FDR-adjusted p; y axis) of temporally regulated sites relative to delta editing levels (x axis). (C) Sites according to the temporal bias are partitioned by genic region. (D) Schematic for estimating miRNA binding affinity to 3′ UTRs and changes in estimated minimum free energy (MFE) with and without A-to-G editing (left). Differences in miRNA MFE computed for only high confident local alignments between miRNA seed regions and 3′ UTRs (right). Significance was tested using a two-sided Mann-Whitney U test. (E) Pairwise Pearson’s correlation of temporal changes in editing levels (delta editing rates) among the DLPFC, cerebrum (n = 55), and cerebellum (n = 59). (F) Median editing levels for sites with an increasing pattern across development (log age, x axis). (G) Functional enrichment of genes harboring a site with an increasing profile and the top 5 enriched categories are depicted. (H) The same genes were examined for enrichment of neurodevelopmental disorder-related genes and gene sets identified from large-scale genetic and genomic studies. (I) Sites with an increasing profile were examined for enrichment of editing sites previously found to be dysregulated in studies of postmortem brain tissue from individuals with neurodevelopmental disorders. Pink line indicates a Benjamini-Hochberg adjusted p < 0.05.

**Figure 4.**
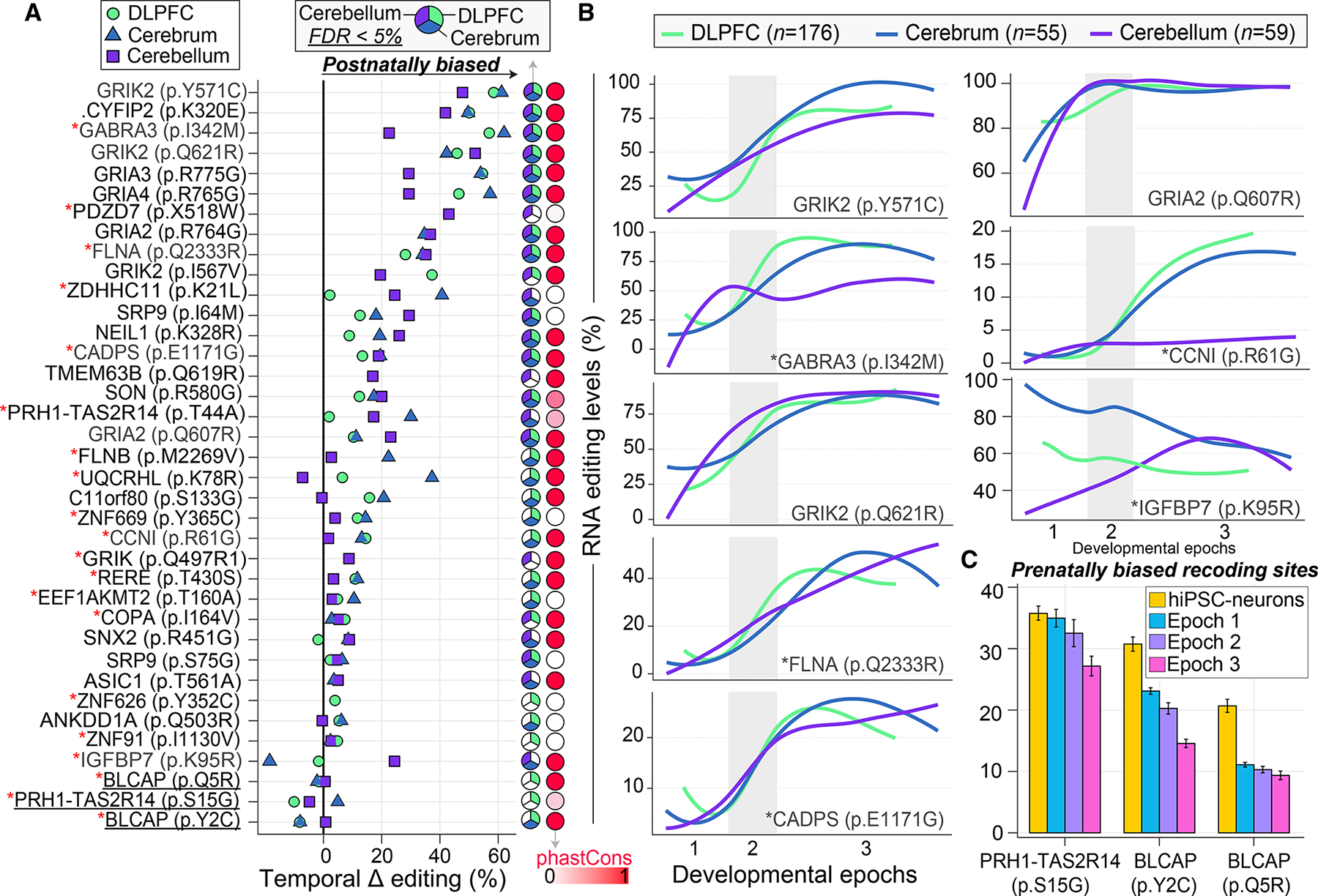
Spatiotemporal dynamics of RNA recoding sites across development (A) Ranking of 37 recoding sites (y axis) by temporal effect sizes (delta editing rates; x axis) between prenatal and postnatal periods. Each recoding site exhibits a significant change in editing levels in at least one anatomical region. Pie charts indicate where a recoding site is significantly temporally regulated (FDR < 5%). PhastCons scores represent probabilities of negative selection and range between zero (white) and one (red). Red asterisks (*) indicate sites that validate in mature hiPSC-derived neurons. (B) Examples for eight spatiotemporally recoding sites with significant changes in editing levels (y axis) across development (log age, x axis) in the DLPFC (n = 176), cerebrum (n = 55), and cerebellum (n = 59). These sites include well-known sites (e.g., GRIK2 [p.Y571C], GRIA2 [p.Q607R]) and other sites with unexplored roles in neurodevelopment. The late fetal transition (epoch 2) is shaded in gray. Loess curves were used to fit the data. (C) Fetal validation of the three prenatal specific recoding sites in mature hiPSC-derived neurons (day 77; n = 30) relative to the DLPFC.

**Figure 5.**
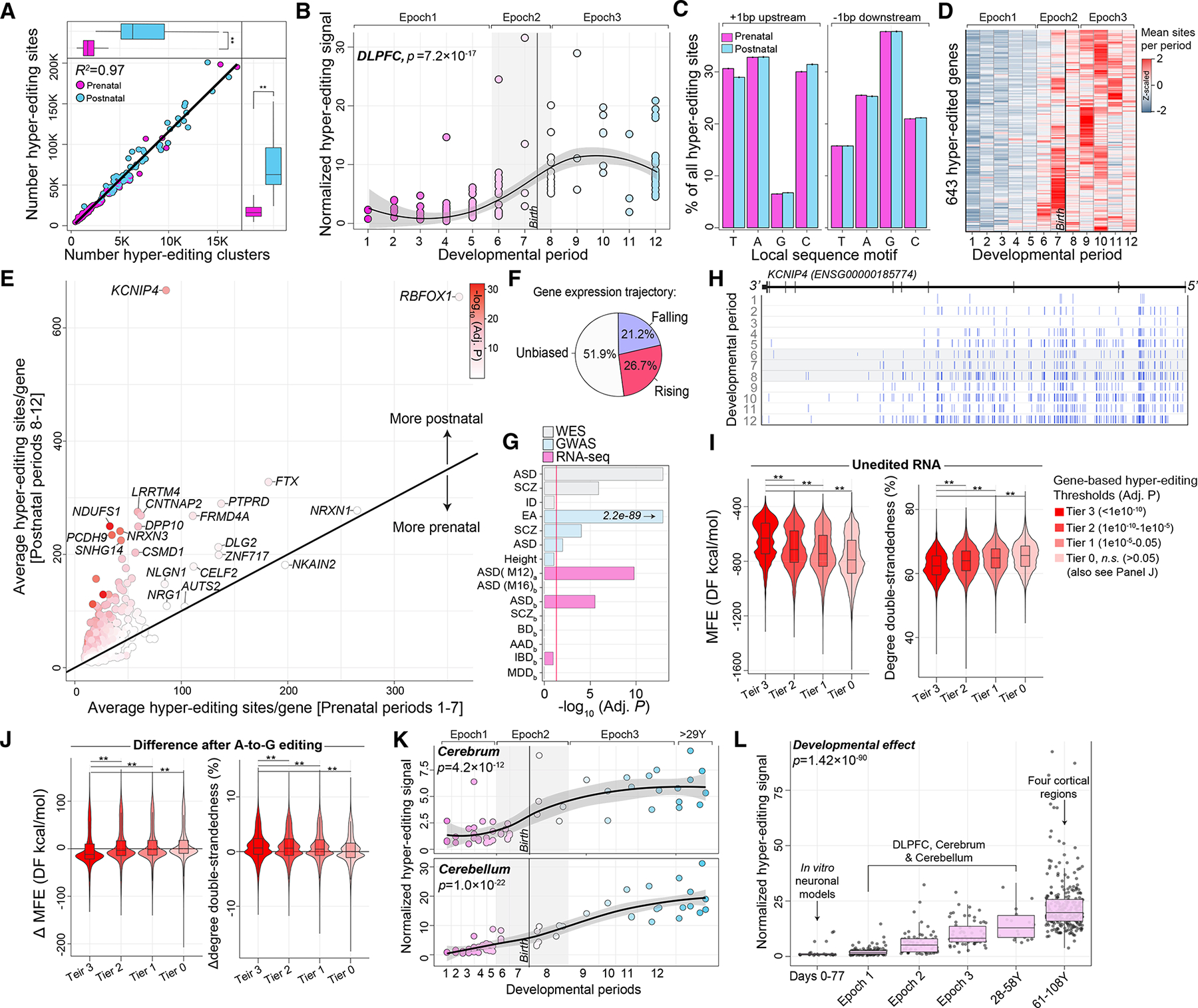
RNA hyper-editing across human brain development (A) Hyper-editing sites in the DLPFC (y axis; n = 176) increase during postnatal development and are concordant with the frequency of hyper-editing clusters (x axis). (B) DLPFC normalized RNA hyper-editing signal (y axis) across development (log age; x axis). (C) Hyper-editing sites enrich for a common local sequence motif. (D) Heatmap of genes that amass hyper-editing events during postnatal development. The number of hyper-editing sites per period are averaged for each gene and z scaled. (E) Mean number of hyper-editing sites per gene during prenatal periods 1–7 (x axis) versus postnatal periods 8–12 (y axis). (F) The developmental expression trajectories for genes that amass hyper-editing sites during postnatal periods. (G) Genes enriched for postnatal hyper-editing enrich for neurodevelopmental disorder genes curated from independent genomic studies. (H) RNA hyper-editing barcode plot illustrates when and where hyper-editing sites amass in *KCNIP4*, an educational attainment gene. (I) Minimum free energy (MFE) and the degree of double-strandedness predictions for RNA secondary structures without hyper-editing. Secondary structures were assigned back to a gene, and genes were parsed according to level of postnatal hyper-editing enrichment to form four tiers of genes. (J) Changes in MFE and degree of double-strandedness following hyper-editing. Two-sided Mann-Whitney U tests were used to test for significance. (K) Normalized hyper-editing levels across cerebrum (n = 55; top) and cerebellar (n = 59; bottom) development. (L) Compiling the normalized hyper-editing signal across development (n = 575), including hESCs (n = 24) and normal aging (n = 261). Two-sided linear regression analyses tested for significance.

To catalog high-confidence selective RNA editing sites, three complementary RNA editing site-calling techniques were utilized followed by a series of comprehensive detection-based thresholds (Figures 2A and S4). In brief, we used two *de novo* callers (REDItools2^40^ and JACUSA2^41^) to uncover high-quality A-to-I sites not already cataloged in editing databases together with a supervised approach^27^ applied to three large lists of known sites^31,42,43^ to ensure ample collection of bona fide A-to-I editing sites across development. In the DLPFC, ~60,951 editing sites were detected per sample (Table S1), which showed consistent hallmark signatures of RNA editing: (1) the vast majority were A-to-G sites (~98% prenatal, ~97% postnatal); (2) mapped to *Alu* repeats (~89% prenatal, ~88% postnatal) (Figure 2A); (3) were predominately known sites cataloged in editing databases (~94% prenatal, ~94% postnatal) (Figure 2A); (4) sites not cataloged in existing databases predominately consisted of A-to-G modifications (~65% prenatal, ~73% postnatal) and were enriched for a common sequence motif whereby guanosine is depleted upstream (−1 bp) and enriched downstream (+1 bp) of the target adenosine, consistent with known A-to-G editing motifs (Figure 2B); (5) sites were mainly located in introns and 3′ UTRs, with a significant enrichment of RNA editing sites detected within 3′ UTRs among postnatal samples relative to prenatal samples (p = 2.7 × 10^−13^) (Figure 2C). Notably, site discovery is largely correlative with sequencing depth and no differences in library depth were observed between prenatal and postnatal DLPFC samples (Figure S5).

Next, we evaluated the overlap of high-quality editing sites detected for each sample relative to all other samples. A convergence of sites consistently detected either during prenatal and/or postnatal periods was uncovered, with the largest shift in convergence occurring during the late fetal transition (Figure 2D). Based on this result, we aggregated editing sites into three main groups: (1) prenatal predominant sites detected only in prenatal samples (n = 3,248); (2) postnatal predominant sites detected only in postnatal samples (n = 7,456); and (3) “common” sites with high detection rates across all DLPFC samples (n = 10,027). Notably, the editing levels for prenatal predominate sites were significantly higher when compared with postnatal predominate sites (p = 3.4 × 10^−112^, ~10% higher, Figure 2E). Most prenatal predominant sites mapped to genes with a falling expression trajectory over development (77.4%), while postnatal predominant sites mapped to genes with a rising expression (54.4%) (Figure 2F).

Validating the accuracy of our approach, the same analytic framework was applied to two independent bulk RNA-seq datasets of cerebrum (n = 55) and cerebellar (n = 59) development. Across the cerebrum and cerebellum, ~22,400 and ~39,209 sites were detected per sample, respectively, and the majority were A-to-G events (~95% in cerebrum, ~97% in cerebellum) and mapped to *Alu* elements (86% in cerebrum, 89% in cerebellum) (Figure S6; Table S1). The cerebellum harbored significantly more sites relative to the cerebrum, irrespective of similar sequencing yields (p = 0.001, 75% increase), and displayed a significant postnatal increase in site detection (p = 0.0005, 115% increase) (Figure S5). Ensuing analyses also confirmed: (1) a significant enrichment of site detection in 3′ UTRs among postnatal samples relative to prenatal samples in both the cerebrum and cerebellum (p = 0.01, p = 0.0001, respectively, Figure 2G); (2) a convergence of RNA editing sites detected during either prenatal and/or postnatal periods (Figure 2H); (3) and prenatal predominate sites were more highly edited compared with postnatal sites (cerebrum, p = 1.2 × 10^−78^; cerebellum p = 1.1 × 10^−17^, Figure 2I). Furthermore, we investigated the concordance of editing levels for prenatal and postnatal predominate sites share between two or more anatomical regions, and confirmed a high level of correlation across regions, validating their developmental biases (Figure S6A). Notably, prenatal and postnatal predominate sites detected in two or more regions generally mapped to 3′ UTR regions and occurred in several developmental genes (Figures S6B and S6C).

### Spatiotemporal changes in RNA editing levels across brain development and disease

Analysis of 10,027 bona fide selective editing sites across all DLPFC samples revealed that developmental period indeed explained the largest fraction of RNA editing level variability (Figures S7A and S7B). Principal components distinguished prenatal from postnatal samples based on editing levels for these sites and PC1 was strongly correlated with developmental age (*r* = 0.82, p = 5.7 × 10^−58^) (Figure 3A). Differential editing identified temporally regulated sites: 8,130 showed higher editing postnatally (“postnatal biased”), 274 prenatally (“prenatal biased”), and 1,623 were constant (“unbiased”) across cortical development (Figure 3B; Table S2). Adjusting for *ADAR* resulted in 2-fold reduction of temporally regulated sites (Figure S8C; Table S2).

Most temporally regulated editing sites increased in editing efficiencies and mapped to 3′ UTRs (Figure 3C). Accordingly, we modeled whether RNA editing in 3′ UTRs might influence miRNA binding efficiency (see STAR Methods) (Table S2). A significant reduction in miRNA binding energy was observed in edited 3′ UTRs relative to un-edited 3′ UTRs (p = 2.3 × 10^−32^, Mann-Whitney U test) (Figure 3D), suggesting a regulatory role for RNA editing in cortical development. Moreover, concordance analysis between RNA editing and corresponding gene expression profiles identified sites in 3′ UTRs as having the largest overall effect and negatively correlated with expression changes across development (Figure S8).

To validate these temporal profiles, we queried editing sites commonly detected across all cerebrum (n_sites_ = 7,155) and cerebellum (n_sites_ = 11,020) samples. Similarly, in the cerebrum and cerebellum, PC1 was positively correlated with developmental age (*r* = 0.89, p = 2.9 × 10^−20^; *r* = 0.94, p = 1.2 × 10^−28^, respectively), most sites were postnatally biased and significantly increased in editing levels (FDR < 5%) (n_sites_ = 4,691, n_sites_ = 6,417, respectively) and mapped to 3′ UTRs (~50%, ~43%, respectively). Again, adjusting for ADAR expression had the largest effect on the number of temporally regulated sites (Figure S9). Temporal changes in editing levels were highly concordant and validated across the DLPFC, cerebrum, and cerebellum (Figure 3E), with a high overlap of significantly temporally regulated sites across all anatomical regions (n_sites_ = 2,126); the vast majority (~99%) were postnatally biased (Figures S10A and S10B). Notably, postnatally biased editing sites derived from the DLPFC were able to accurately predict prenatal from postnatal samples in the cerebrum and cerebellum (Figures S10C and S10D), indicating that sites with increasing editing levels across development are highly spatially conserved (Figure 3F). Functional annotation of genes harboring postnatally biased editing sites implicates transcription and translation regulation, mRNA binding, AMPA receptors, actin cytoskeleton, and metabolism-related terms (Figure 3G), and the sites were generally localized in the postsynaptic density (Figure S11). Postnatally biased editing sites were also enriched on genes associated with neurodevelopmental disorders (Figure 3H). Moreover, A-to-I sites previously reported to be disrupted in postmortem brain tissue from donors with autism spectrum disorder^44^ and schizophrenia^27^ are also temporally regulated and show an increasing pattern of editing across development (Figure 3I).

### Spatiotemporal regulation of RNA recoding sites

RNA recoding sites are those in protein-coding regions, which change amino acid sequences. We identified 37 recoding sites that significantly change in editing levels throughout prenatal and postnatal development in at least one brain region. Of these, 31 significantly changed in at least 2 regions and 28 are highly evolutionarily conserved (Figure 4A). Most temporally regulated recoding sites increased in editing levels across development, with only three sites showing a general prenatal bias (p.Y2C and pQ5R in *BLCAP*; p.S15G in *PRH1-TAS2R14*). All together, these recoding sites mapped to 31 unique genes enriched for ionotropic glutamate receptor activity (FDR = 1.46 × 10^−8^), ligand-gated ion channel activity (FDR = 4.95 × 10^−8^), AMPA glutamate receptor activity (FDR = 2.27 × 10^−7^), and neuronal projection (FDR = 9.38 × 10^−5^). Ranking these sites by temporal effect sizes confirmed several functional recoding sites known to increase editing levels through development, including those mapping to a collection of excitatory, inhibitory, and G-coupled protein receptors (e.g., *GRIK2*, *GABRA3*, and *GRIA2*). However, we also highlight many recoding sites with undefined roles in neuronal development: p.R61G site in cyclin I (*CCNI*), peaking postnatally in the cortex but not in the cerebellum; p.K95R site in insulin-like growth factor binding protein 7 (*IGFBP7*), peaking postnatally in the cerebellum but prenatally in the cortex; p.I64M and p.S75G sites in signal recognition particle 9 (*SRP9*) peaking postnatally across all regions; a postnatal biased p.R580G site in SON DNA and RNA binding protein (RBP) (*SON*, the cause of ZTTK syndrome), among others (Figures 4A and 4B). Importantly, 20 recoding sites were validated by quantifying their editing levels in mature hiPSC-derived neurons (day 77), which illustrate editing levels comparable with those during fetal development (Table S3). For the three prenatally biased recoding sites, we confirmed higher editing levels in mature hiPSC-derived neurons relative to both fetal and postnatal DLPFC tissue (Figure 4C).

### RNA hyper-editing increases throughout development and stabilizes secondary structures

Unmapped RNA-seq reads from the DLPFC were used to quantify RNA hyper-editing^21,45^ for several modification types, and A-to-G editing accounted for ~99% of substitutions (Figure S12). Hyper-editing in 3′ UTRs and introns comprised ~80% of all hyper-edited sites with very few in coding regions (~0.24%) (Table S4). Notably, few selective editing sites detected from mapped bam files resided within the identified hyper-edited regions (Figure S13), supporting a separate analysis of these events. The rate of hyper-editing significantly increased from prenatal (μ = 24,428 sites) through postnatal periods (μ = 78,441 sites) (p = 1.4 × 10^−17^) (Figure 5A). To minimize technical variability and enable a direct comparison of hyper-editing across development, we computed a normalized the rate of hyper-editing to the number of mapped bases per million per sample. Normalized hyper-editing signal increased in frequency into postnatal periods (p = 7.2 × 10^−17^, Cohen’s d = 3.06) (Figure 5B). These rates were associated with the proportion of mature neurons (R^2^ = 0.41) and *ADAR2* expression (R^2^ = 0.21) (Figure S13) and remained significantly postnatally biased following adjustment for neuronal proportions (p = 1.0 × 10^−11^). Local sequence motifs were consistent with the expected distribution of guanosines 1 bp (±) the target adenosine (Figure 5C).

Next, we identified 643 genes that amassed a significant number of hyper-editing sites throughout development after adjusting for neuronal proportions and gene length (Figures 5D and 5E, Table S4). Approximately 74% displayed expression profiles that were either falling or non-transient over development, suggesting that hyper-editing enrichment is independent of corresponding expression levels (Figure 5F). Genes that accumulate hyper-editing during postnatal development are enriched for transsynaptic signaling, Ca^2+^ signaling, protein kinase activity, and ion channel regulator activity (Table S4). These genes are further enriched for loci implicated in risk for autism, educational attainment, and schizophrenia, as well as genes that are significantly differentially expressed in the cortex of individuals with autism (Figure 5G). For example, gradual accumulation of hyper-editing sites was observed in the first and second introns of potassium voltage-gated channel interacting protein 4 (*KCNIP4*) (Figure 5H).

To complement these results, minimum free energy (MFE) and degree of RNA double-strandedness was calculated for each hyper-edited region (±1,000 bp each cluster boundary). Investigating the natural unedited primary sequences for each region revealed an elevated MFE and reduced double-strandedness specifically for regions and transcripts that significantly accumulate hyper-editing sites during postnatal development (Figure 5I). Thus, genes enriched for postnatal hyper-editing are less stable than other regions and transcripts in the absence of hyper-editing. Next, investigating the hyper-edited sequences revealed that regions and transcripts that gain hyper-editing postnatally exhibit the greatest reduction in MFE and increase in double-strandedness relative to regions that do not undergo significant postnatal hyper-editing (Figure 5J). Thus, temporal hyper-editing may act as a timely mechanism to selectively stabilize secondary structures for specific RNA transcripts.

Given that most hyper-editing sites accumulate in non-coding regions of neurodevelopmental genes, we also tested whether these sites are predicted to be splice altering and/or occur in retained introns (Table S4). First, the number of hyper-editing sites predicted to cause cryptic splicing increased across development (p = 2.9 × 10^−19^) yet accounted for only a small fraction of the total number of sites (<1%) (Figure S14). Second, the number of uniquely detected introns increased postnatally (p = 0.0006), and ~80% of genes exhibiting hyper-editing in introns also displayed increased intron retention (IR) during postnatal development (Figure S14); notably the rate of IR did not correlate with gene expression.

To confirm and validate these profiles, we reproduced a significant postnatal bias in the normalized RNA hyper-editing signal throughout cerebrum (p = 4.2 × 10^−12^, Cohen’s d = 2.82) and cerebellum development (p = 1.0 × 10^−22^, Cohen’s d = 2.43) (Figure 5K), and validated the postnatal enrichment of hyper-editing sites on a per gene basis across both anatomical regions (Figure S15A). Next, we computed the RNA hyper-editing signal using *in vitro* hESCs and again confirmed a significant increase throughout neuronal maturation (p = 0.007) (Figure S15B). Notably, hyper-editing was significantly elevated during pluripotency (μ = 21,796 sites) relative to remaining days post neural induction (μ = 2,163 sites, p = 1.9 × 10^−9^) (Figures S15C–S15F). In the following, we show that, while the hyper-editing rate was not dynamically regulated during advanced aging (Figure S16), we validate the majority of genes harboring an enrichment of postnatal hyper-editing sites with consistent direction of effect (R^2^ = 0.58) (Figure S16; Table S4). Finally, we compiled results across all datasets and uncovered a steady increase in the normalized hyper-editing signal throughout all stages of neuronal maturation and brain development, peaking into advanced stages of aging (p = 1.4 × 10^−90^) (Figure 5L).

### Temporal increases in A-to-I editing are a conserved feature of mammalian brain development

**Figure 6.**
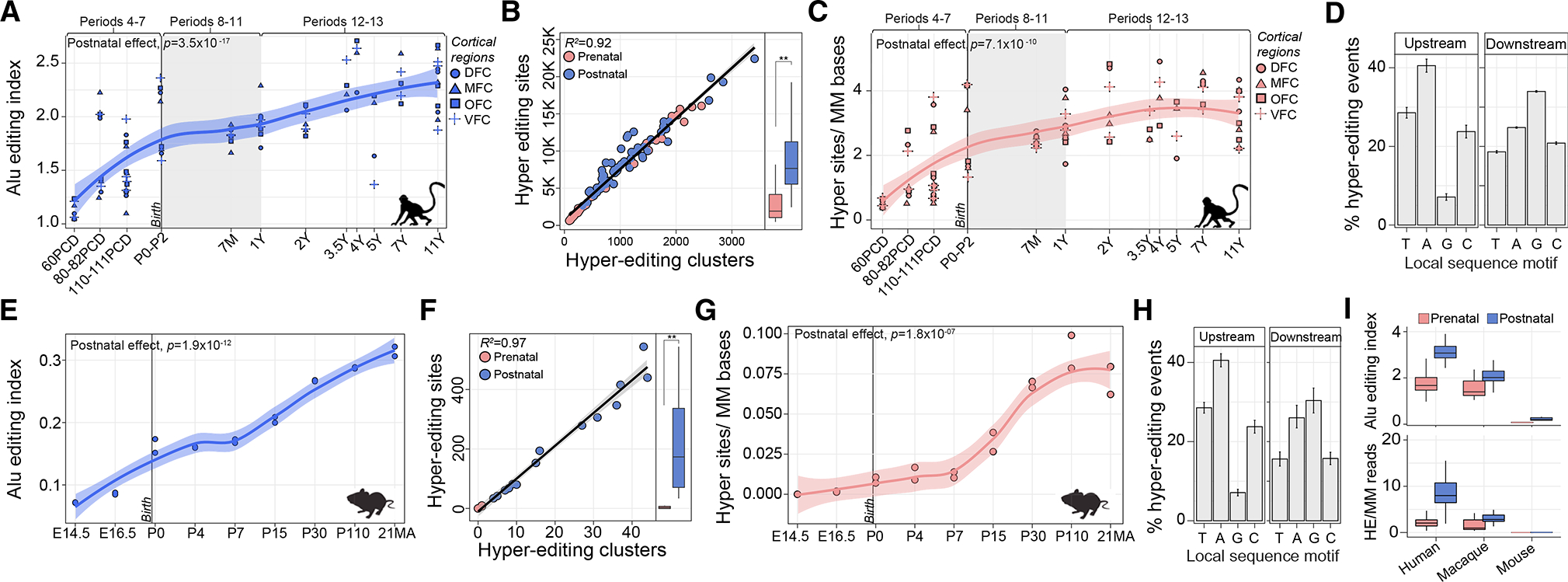
Temporal dynamics of RNA editing in animal models of neurodevelopment (A) The AEI (y axis) of four cortical regions (DFC, MFC, OFC, and VFC) across rhesus macaque (n = 26 biological replicates) development (log age; x axis). Macaque developmental periods were matched with those closest to human as described previously.^37^ (B) Hyper-editing site detection (y axis) and the number of hyper-editing clusters (x axis). The number of hyper-editing sites increases into postnatal development.**p = 2.8 × 10^−6^. (C) Temporal increase in normalized hyper-editing levels across development. p = 7.1 × 10^−7^. (D) Local sequence motifs for hyper-editing sites 1 bp upstream and downstream of the target adenosine (standard error bars represent sample level variability). (E) The AEI (y axis) of whole cortex in mouse (n = 18) across nine developmental periods (x axis). (F) Hyper-editing site detection (y axis) and the number of hyper-editing clusters (x axis). The number of hyper-editing sites increases into postnatal development. **p < 2 × 10^−16^. (G) Temporal increase in normalized hyper-editing levels across developmental periods. (H) Local sequence motifs for hyper-editing sites 1 bp upstream and downstream of the target adenosine (standard error bars represent sample level variability). (I) Compiling the AEI and normalized hyper-editing levels across prenatal and postnatal stages between humans (using DLPFC; n = 176), rhesus macaque and mouse. Linear regression was used to compute significance for all tests.

To test if these temporal profiles constitute a conserved regulatory mechanism, we repeated these analyses leveraging two animal models of cortical development: (1) bulk tissue RNA-seq of four cortical regions of 26 rhesus macaques (60 post-conception days to 11 years); (2) whole-cortex RNA-sequencing of 18 wildtype mice (E14.5 to postnatal 21 months) (Table S5). In macaque, we observed a significant postnatal bias in the AEI (p = 3.5 × 10^−17^) and in normalized hyper-editing signal (p = 7.1 × 10^−10^) (Figures 6A–6C). Hyper-editing clusters ranged in size from 65 to 72 bp in length, each contained ~7.8 hyper-editing sites, and all sites shared a local sequence motif similar to that observed in humans (Figure 6D). These findings also reproduced in mouse, including an increased AEI (p = 1.9 × 10^−12^), normalized RNA hyper-editing signal (p = 1.8 × 10^−7^), and conservation of common local sequence motif for all hyper-editing sites (Figures 6E–6H). Finally, we observed that the AEI and the frequency of hyper-editing sites are highest in human cortex, followed by macaque and subsequently mouse (Figure 6I). Importantly, all hyper-editing sites in the current study were robust to potential false positives and not confounded by common genomic variation (Figures S12D and S12E).

### Temporal *cis*-edQTLs and colocalization with neurological traits and disorders

**Figure 7.**
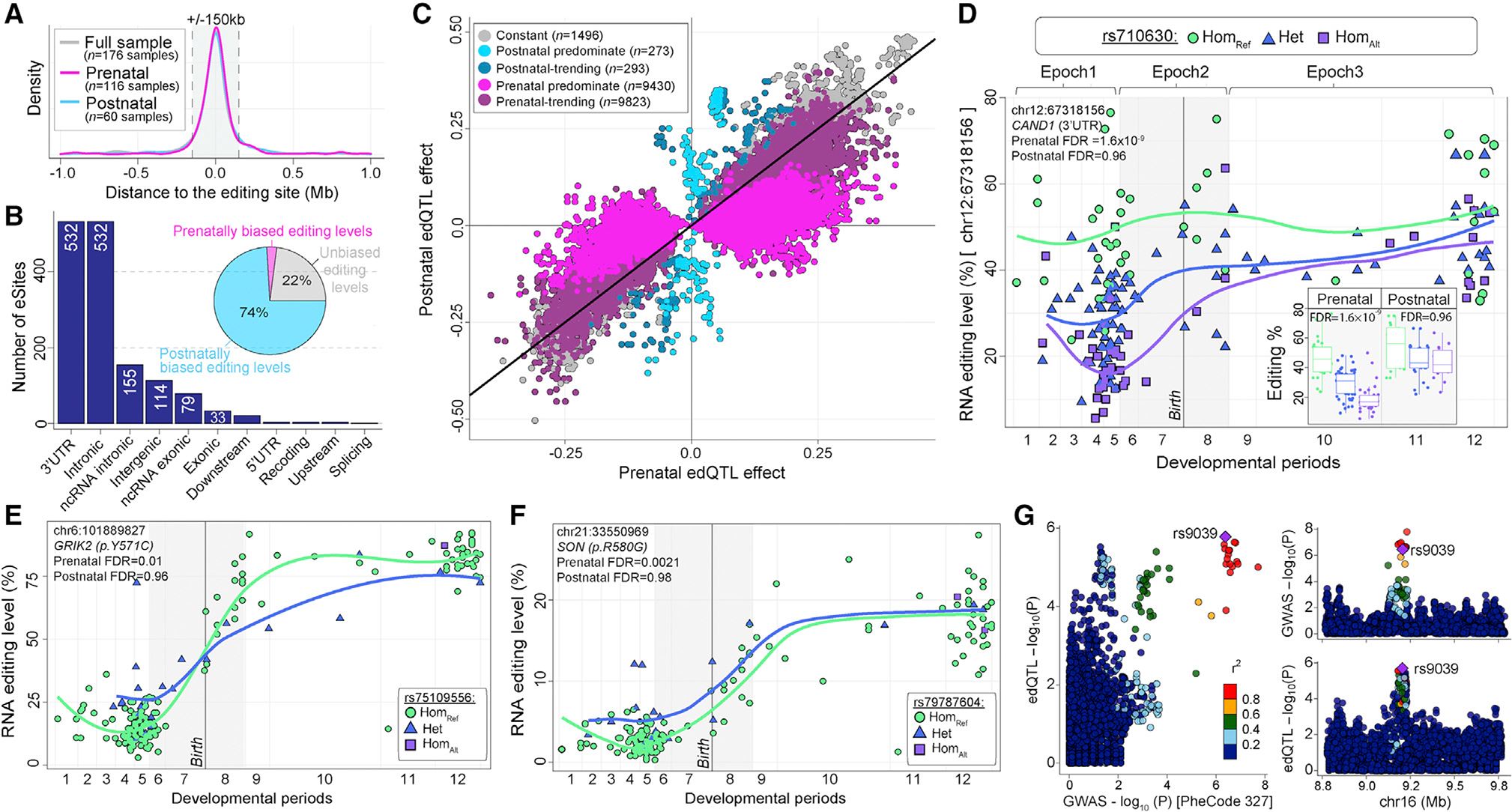
Temporal predominate *cis*-edQTLs in the dorsolateral prefrontal cortex (A) Distribution of the association tests in relation to the distance between the editing site and variant for max-edQTLs. The gray box indicates ±150 kb relative to the editing site. (B) eSites parsed by genic region and temporal editing levels in the DLPFC (inset pie chart). (C) Prenatal (x axis) versus postnatal (y axis) effect sizes for all significant edQTLs. edQTLs are split into five categories based on temporal predominance using effect size and statistical thresholds. (D–F) (D) RNA editing levels binned by genotype for a top prenatal-predominant edQTL for *CAND1*. Curves were fit using loess trajectories for RNA editing levels in samples with each of three genotypes. Inset boxplots for prenatal (left) and postnatal (right) periods with each of three genotypes. Example of prenatal predominate edQTLs for two recoding sites in (E) *GRIK2* (p.Y571C) and (F) *SON* (p.R580G). Lines represent loess trajectories for RNA editing in samples with each genotype. (G) Locuscompare plots of the top co-localized hit for variant rs9039 associated with sleep disorders (PheCode 327).

WGS data were used to detect SNPs that could influence RNA editing levels (edQTL, editing quantitative trait loci). RNA editing levels were fit to SNP genotypes, covarying for developmental period, sex, and the first five principal components of ancestry, as well as *ADAR1* and *ADAR2* expression (see STAR Methods). To distinguish temporal-predominant edQTLs, we performed three *cis*-edQTL analyses, leveraging: (1) prenatal samples only (n = 116, periods 1–7); (2) postnatal samples only (n = 60, periods 8–12); and (3) all samples (n = 176, periods 1–12). We defined a 1 Mb window (±) to search for SNP-editing pairs of an editing site and identified 31,324 *cis*-edQTLs from all samples, 20,659 *cis*-edQTLs from prenatal samples, and 2,066 *cis*-edQTLs from postnatal samples at FDR < 5% (Table S6). These edQTLs comprised a total of 1,039, 790, and 164 unique editing sites (eSites) across all samples, prenatal samples, and postnatal samples, respectively. Each lead SNP was located close to their associated editing site (±150 kb) (Figure 7A). eSites were generally increasing in editing levels throughout development (~75%) and mapped to 3′ UTRs and introns, with fewer in exonic regions (n = 34) (Figure 7B).

The majority of edQTLs were prenatal-predominant, with greater prenatal than postnatal effect sizes, while fewer edQTLs were postnatal-predominant, with significantly greater postnatal than prenatal effect sizes (Figure 7C). Approximately 40% of prenatal-predominant edQTLs occur with eSites mapping to 3′ UTRs. An example of a prenatal-predominate edQTL, featuring an A-to-I site in the 3′ UTR of the *CAND1* gene (Figure 7D). We also observed prenatal predominate edQTLs for two recoding sites, one in *GRIK2* (p.Y571C) and another in *SON* (p.R580G), whereby heterozygous genotypes result in higher fetal editing levels for these sites, which later equalize during postnatal development (Figures 7E and 7F). With increased sample sizes, we expect a larger fraction of edQTLs to show some degree of temporal specificity, especially postnatal. While the magnitude of effect varied across development for many edQTLs, we did not observe a single edQTL with directly opposing prenatal and postnatal directions of effect.

Finally, we queried edQTLs for co-localization with common genetic risk variants for CNS traits and disorders by leveraging summary statistics from several genome-wide association studies (GWAS) (see STAR Methods). We found weak co-localization (*PPH4* 0.4–0.8) for 23 loci across 21 traits and disorders and strong evidence of co-localization (*PPH4* > 0.8) for 2 loci across 2 disorders (Table S6). Disease variants uniformly colocalized with postnatal predominant edQTLs (n = 7) followed by consistent (n = 13) and prenatal predominant edQTLs (n = 16). The majority of colocalization occurred with editing events in 3′ UTRs. The strongest colocalization occurred between a common genetic variant for sleeping disorders and an edQTL in the first non-coding exon of *C16orf72* (*PPH4* = 0.98) (Figure 7G).

## DISCUSSION

As the catalog of RNA editing sites in the brain continues to grow, the physiological relevance for most sites remains unknown. Because A-to-I editing sites with significant functional effects are known to be tightly temporally regulated,^1,24,25^ elucidating additional spatiotemporally regulated sites across neurodevelopment, and those that are genetically regulated, allows further characterization of potentially functional mechanisms of brain development. These results provide an atlas of such sites and reveal several important aspects of RNA editing throughout development: (1) global *Alu* editing significantly increases throughout brain maturation, and this pattern is evolutionarily conserved in mammalian models of neurodevelopment; (2) underlying this shift, thousands of temporally regulated sites were uncovered that are spatially conserved and increase in editing levels across development, the majority in 3′ UTRs of essential neurodevelopmental genes, which are predicted to stabilize local miRNA interactions more so during postnatal development; (3) the minority of spatiotemporally regulated sites occur in protein-coding regions, and a total of 37 RNA recoding sites show significant changes in editing levels across development, including many with known and unknown functional effects; (4) hyper-edited regions amass during advanced stages of aging, and are predicted to have diverse regulatory effects; (5) thousands of sites illustrate either prenatal and/or postnatal predominate edQTLs, the majority of these sites occur in non-coding regions, although few recoding sites also exhibit significant prenatal edQTLs. Collectively, these findings establish starting lines for investigations to link RNA editing with mechanisms of brain maturation. We discuss these points in turn below.

The most significant temporal changes in global *Alu* editing occur during the late-fetal transition, between mid-fetal development and infancy (Figure 1), consistent with timing in gene expression patterns.^32^ Reports in humans and primates suggest that this transitional period reflects alterations of both cell types and molecular processes within these cells.^32,33,37^ Notably, temporal shifts in *Alu* editing were synchronized with, but not fully explained by, variation in *ADAR2*. This was similarly modeled in *C. elegans*, whereby neurodevelopmental expression changes permit ADR-2 to bind more efficiently to neural transcripts, resulting in increased editing.^46^ In humans, the AEI and *ADAR2* is elevated in neurons compared with glial cells.^31^ Nevertheless, significant temporal increases in the AEI remained after adjusting for neuronal content, on par with previous observations.^24,25^ Indeed, temporal variation in *Alu* editing most likely arises through a combination of discrete regional- and cell-type-specific regulation of ADAR enzymes and target transcripts that act in a temporal pattern. Broadly, our results support a model whereby unedited transcripts are transcribed, and perhaps translated, more predominately during fetal periods of development, whereas the edited transcripts are more abundant in the adult brain. This trend is also conserved in primate and murine models, suggesting a general restraint on editing during early mammalian brain development with functional consequences for brain maturation. The enzymatic activity of *ADAR2* most likely plays a critical role in this process. RBPs may also lend to the temporal changes in *Alu* editing, as they are highly expressed during fetal development^32,47^ and could drive global editing levels downward during prenatal periods by outcompeting ADAR enzymes for dsRNA structures. Indeed, RBPs interact with ADARs and act as negative regulators of RNA editing activity^39,46,48^ but have yet to be dissected in human neurodevelopment.

Many spatiotemporally regulated selective editing sites occur in 3′ UTRs (Figure 3). These results are in line with a previous report of A-to-I editing in brain maturation.^26^ Furthermore, A-to-I editing on 3′ UTRs was predicted to stabilize the duplex formed between miRNAs and their target seed regions during postnatal development. The frequency of A-to-I sites detected on 3′ UTRs was also significantly higher during postnatal periods relative to fetal periods, and this too is spatially conserved. These results bolster earlier work that showed that miRNAs form stronger interactions with precisely edited 3′ UTR substrates^26^ and further illustrate that miRNAs also have a larger cellular pool of such targetable templates available to fine-tune gene expression during postnatal development. Correlation coefficients between temporally regulated gene expression and editing levels in 3′ UTRs are generally negative and differ relative to sites in other genic regions, further supporting A-to-I editing in 3′ UTRs as a regulatory mechanism affecting RNA abundance throughout brain development. Indeed, sites with increasing editing patterns occur on genes implicated in transcriptional and translational regulation, mRNA binding, and metabolism. Importantly, these sites, among other spatiotemporally regulated sites, are commonly disrupted in postmortem brain tissues from individuals with autism spectrum disorder^44^ and schizophrenia,^27^ providing immediate avenues for dissecting their pathological impacts through altered miRNA binding and regulation of gene expression in *trans*.

Beyond non-coding regions, 37 spatiotemporally regulated recoding sites were uncovered (Figure 4). Each recoding site exhibits different maximum editing levels during postnatal development, ranging from ~15% to 100% and underscore their precise regulation. These results confirm well-known functional sites that regulate Ca^2+^ permeability (*GRIA2*, p.Q607R, ~20% increase; *GRIK2*, p.Y571C, ~58% increase),^10–12^ remodel actin cytoskeleton at excitatory synapses (*CYFIP2*, p.K320E, ~50% increase),^13,14,49^ and guide gating kinetics of inhibitory receptors (*GABRA3*, p.I342M, ~56% increase).^15,16^ Analyses identified over 20 other highly conserved spatiotemporally recoding sites where the functional implications remain less clear and warrant deeper investigation. We outline five such examples here. First, the p.Q2333R site in *FLNA* increases ~27% through brain development. This site was recently found to be a regulator of cytoskeletal organization and cell mechanics.^50^ The temporal profile of this site suggests that it may increase actin crosslinking and reduce cell migration during postnatal brain development. Second, a ~15% increase in the p.R61G site in cyclin-I (*CCNI*) was observed in the cerebrum but not in the cerebellum. This site resides in the cyclin N-terminal domain of the protein and has been identified to be highly edited in GABAergic neurons,^31^ where, like its encoded gene, this site may support a role in the cell cycle by activating cyclin-dependent kinases.^51^ Third, a ~20% increase in the p.E1171G site in calcium-dependent secretion activator (*CADPS*) was observed, a site with high editing levels reported in glumatergic neurons.^31^ Increased editing at this site has been shown to increase expression and synaptic localization of *CADPS* and enhance short-term synaptic plasticity,^52^ which may have significant neuronal network effects throughout brain development. Fourth, divergent temporal editing profiles were observed for p.K95R in insulin-like growth factor-binding protein 7 (*IGFBP7*), a site that decreases ~25% in the cortex but increases ~25% in the cerebellum across development. This site is in the heparin-binding site of *IGFBP7* and is also part of the recognition sequence for proteolytic cleavage. Thus, A-to-I editing may act as a regional-specific mechanism that influences heparin binding and/or proteolytic processing and its downstream effects regarding apoptosis, regulation of cell growth, and angiogenesis.^53^ This site is also temporally regulated during aging in porcine brain tissues.^54^ Fifth, recoding site p.T430S in arginine-glutamic acid dipeptide repeats (*RERE*) increases ~10% across development. RERE is crucial for regulating apoptosis and retinoic acid signaling during embryonic development.^55,56^ Collectively, these sites are all conserved and increase in editing levels across development, like well-known functional sites.^1,10–16,49^ Collectively, these results feature numerous entry points for functional and mechanistic interrogation of recoding sites throughout neurodevelopment.

Extended regions of hyper-editing also increase in frequency through development and amass in the brain during advanced stages of aging (Figure 5). These regions are located most often in *Alu*-rich regions and within introns and 3′ UTRs that form dsRNA structures, consistent with previous reports.^21,45,57^ These events occur in hundreds of genes involved in transsynaptic signaling, ion channel regulator activity, and those implicated in neurodevelopmental disorders. Still, deciphering how hyper-editing influences gene function remains challenging, especially for hyper-editing within introns, which is therefore limited to premRNA. Hyper-edited transcripts can be subjected to nuclear retention or degradation^58,59^ and have been found to transiently interact with components of cytoplasmic stress granules.^60,61^ Hyper-editing has also been shown to suppress interferon induction and apoptosis, suggesting that these transcripts play a role in the stress response^62^ but require further study in neurodevelopment and aging. To compliment these findings, we probed additional functional consequences of hyper-editing in the developing brain. First, and in very rare cases, hyper-editing near exon-intron boundaries could cause cryptic splicing, subsequently accounting for a tiny fraction of observed isoform diversity. Second, and more generally, hyper-editing could alter the local dsRNA structure, which, in turn, will have an impact on gene expression and protein abundance. We estimated that hyper-editing increases the stability of RNA transcripts. This stabilizing effect is more pronounced for transcripts that naturally (in an unedited state) exhibit higher MFE and reduced RNA double-strandedness. MFE has been regarded as one of the most reliable predictions of RNA secondary structures,^63^ and RNA editing has been shown to stabilize secondary structures of RNAs in other non-CNS cell types.^64,65^ Indeed, enhanced RNA secondary structure has been linked with increases in mRNA half-life and protein abundance.^66^ Notably, RNA transcripts that accumulate postnatal hyper-editing events are implicated in synaptic signaling, a process that initiates and unfolds throughout postnatal development. Our findings propose a model whereby temporal hyper-editing could serve as a mechanism to ensure stability and function of select synaptic genes throughout postnatal brain maturation. Furthermore, it is plausible that hyper-editing also acts as a mechanism to create or disrupt RNA binding domains for RBPs, yet such data are currently lacking to test this hypothesis in the human brain and related cell types. Finally, it is worth noting that in non-human species, hyper-editing has been linked with social behavior,^67,68^ adaptation to temperature,^69,70^ and other physiological conditions.^71^

Temporal-predominate edQTLs illustrate a greater effect on editing levels prenatally or postnatally (Figure 7). edQTLs can explain upward of ~40% of differences in editing levels within a developmental window for thousands of sites. The majority of edQTLs occur in 3′ UTRs and other non-coding regions, consistent with previous edQTL work by us and others.^27–31^ For the minority of edQTLs in coding regions, two prenatal predominate edQTLs are identified: (1) p.Y571C in *GRIK2*, which may be involved in additional regulation of calcium permeability, together with the p.Q621R site in the same gene^10–12^; and (2) p.R580G in *SON*, which may modulate the pre-mRNA splicing function of *SON*.^72,73^ Such temporal edQTLs highlight unique entry points for the establishment of hiPSC neuronal model systems to further dissect the regulatory mechanisms and functional implications of these recoding sites. For the majority of edQTLs in non-coding regions, unique relationships have been identified between edQTLs, eQTLs, and miRNA expression profiles,^28^ whereby miRNAs can generate an eQTL signal from an edQTL locus via miRNA-mediated transcript degradation. This model has been proposed as a mechanism that alters steadystate transcript levels, linking edQTLs to eQTLs and complex traits. To this end, we also postulated that edQTLs may represent a biologically important intermediate link between genetic variation and disease phenotypes. Yet, edQTLs in the current study rarely co-localized with risk variants for neurological disorders. The most significant colocalization occurred between a variant for sleeping disorders and an editing site in the first non-coding exon of *C16orf72*, previously linked to cognition, learning, and sleeping difficulties.^74,75^ This editing site occurs in a non-coding regulatory region that has been shown to modulate mRNA stability and protein abundance.^76^ To further dissect such mechanisms and their implications for brain maturation and disease risk, it will be critical for future work to greatly increase sample sizes.

### Limitations of the study

This work presents a spatiotemporally resolved atlas of RNA editing in the human brain with extensive *in silico* confirmation and functional predictions; however, a limitation of this work is the lack of experimental validation. Moreover, while our analyses indicate that RNA editing serves as a mechanism that mediates miRNA binding, protein function, mRNA stability, and splicing during critical developmental periods, functional validation is warranted at each of these scales. As such, we present these findings as a resource to springboard future functional investigations of RNA editing in mammalian neuronal maturation, brain development, aging, and genetic disease. Furthermore, without brain cell-type-resolved data across temporal scales, the interpretation of increased RNA editing deserves caution. Indeed, RNA editing activity is higher in neuronal compared with non-neuronal cells and mature neurons increase in frequency across brain development. Thus, our analyses controlled for the proportion of mature neurons and together suggest that this increasing pattern is likely due to higher editing activity per cell during postnatal periods, but this has yet to be studied in a systematic way. Finally, cross-species comparisons indicate higher editing rates in human and non-human primates relative to murine models. *Alu* elements are primate-specific repeats and most editing in mice occurs in B1 and B2 SINE elements, therefore direct comparisons are challenging.

## STAR★METHODS

### RESOURCE AVAILABILITY

#### Lead contact

Further information and requests for resources and reagents should be directed to and will be fulfilled by the lead contact, Michael S. Breen (michael.breen@mssm.edu).

#### Materials availability

This study did not generate new unique reagents.

#### Data and code availability

- All RNA-seq data have been deposited at GEO, synapse, NCBI Short Read Archive and ArrayExpress, and are publicly available as of the date of publication. Accession numbers for each dataset are listed in the key resources table.
- All original code has been deposited at Zenodo and is publicly available as of the date of publication. DOIs are listed in the key resources table.
- Any additional information required to reanalyze the data reported in this paper is available from the lead contact upon request.

### EXPERIMENTAL MODEL AND SUBJECT DETAILS

#### Dorsolateral prefrontal cortex neurodevelopment

A total of 176 paired-end (100bp) samples of human DLPFC covering 12 distinct prenatal and postnatal developmental periods were obtain from synapse (syn21557948).^32^ Processed paired whole-genome sequencing data were also downloaded. The frontal cerebral wall was assayed in nine brains prior-to ten post-conception weeks.

#### Cerebrum and cerebellum neurodevelopment

A total of 114 single-end (100 bp) samples from cerebrum (*n* = 55) and cerebellar (*n* = 59) tissues sampled across prenatal and postnatal periods (4 PCWs-59 + years) were downloaded from ArrayExpress (E-MTAB-6814).^33^

#### Normal aging

A total of 261 single-end (100 bp) samples from the Mount Sinai Brain Bank (MSBB) covering four cortical regions, including BM10, BM22, BM36 and BM44 were downloaded from synapse (syn7416949).^36^ This subset of samples was analyzed as they were largely free of plaque and tangle neurocognitive pathologies and were aged 61–108 years.

#### Human embryonic stem cell corticogenesis

A total of 24 single-end (50 bp) samples of human cerebral cortex development (CORTECON) from human embryonic stem cells (hESCs) across nine time-points (days 0, 7, 12, 19, 26, 33, 49, 63, and 77) were downloaded from GEO (GSE56796).^34^

#### Human induced pluripotent stem cell corticogenesis

A total of 127 paired-end (100 bp) samples from a human induced pluripotent stem cell (hiPSC) neuronal differentiation time course were downloaded from GEO (PRJNA596331).^34^ This dataset covers early differentiating cells (days 2–9), neural progenitor cells (day 15), assembled neuroepithelial rosettes (day 21) and more differentiated neurons (days 49–77).

#### Rhesus macaque neurodevelopment

A non-human primate model (*Macaca mulatta*) of prenatal and postnatal cortical development was downloaded from GEO (PRJNA448973),^37^ covering 96 single-end (75bp) samples, comprising 26 unique donors and four prefrontal cortical areas: MFC – medial prefrontal cortex; OFC – orbital prefrontal cortex; DFC – dorsolateral prefrontal cortex; VFC – ventrolateral prefrontal cortex.

#### Mouse neurodevelopment

A murine model (*Mus musculus*) of prenatal and postnatal whole brain development was downloaded from the NCBI Short Read Archive (SRP055008),^38^ including 18 paired-end (200 bp) cortical samples at nine time points: embryonic day 14.5 (E14.5), E16.5, postnatal day 4 (P4), P7, P17, P30, 4 months, and 21 months.

### METHOD DETAILS

The current study leveraged seven existing large developmental transcriptome datasets and studied the spatiotemporal and genetic regulation of RNA editing across brain development using *in silico* methods and statistics, described below. All reagents and methods were described in the original publications.

### QUANTIFICATION AND STATISTICAL ANALYSES

#### Mapping short-read RNA-sequencing data

STAR^77^ v2.7.3 was used to perform all short-read mapping to reference genome builds for human (hg38), rhesus macaque (rheMac8-Mmul8) and mouse (GRCm38-mm10) in the current study. STAR produced a coordinated-sorted mapped BAM file and unmapped FASTQ file for each sample. The mapped BAM files were used to quantify selective RNA editing sites and the unmapped FASTQ files were used to quantify RNA hyper-editing sites and regions.

#### RNA editing site detection and annotation

A comprehensive RNA editing site detection pipeline was applied to all human DLPFC, cerebrum and cerebellum transcriptome samples. Short-read mapping with STAR produced a coordinate-sorted BAM file of mapped reads, including those spanning splice junctions. High-quality RNA editing sites were quantified from sorted mapped bam files using a combination of two de *novo* callers and one supervised approach:

1. *De novo* caller REDItools v2.0^40^ was applied with the following parameters: -S -s 2 -ss 5 -mrl 50 -q 10 -bq 20 -C -T 2 –os 5. All analyses considered read strandedness (-s) when appropriate.
2. *De novo* caller JACUSA2^41^ with the following parameters: -p 10 –a D, M, Y, E, -m 20. All analyses considered read strandedness when appropriate.
3. A supervised approach queried nucleotide coordinates for all known RNA editing sites using samtools mpileup function, as previously described.^27,31^ This approach was applied to query known RNA editing sites cataloged through REDIportal,^42^ A-to-I sites cataloged across human brain cell types,^31^ and an extensive list of human RNA recoding sites.^43^

Subsequently, filtering steps were applied to retain only high-quality, high-confident bona fide RNA editing sites. The following sites were removed from all analyses: i) all multi-allelic events; ii) all sites mapping to homopolymeric regions or black listed genomic regions in the genome^91^; iii) all sites mapping to common genomic variation in dbSNP(v150) and those in gnomAD with minor allele frequency greater than 0.05; iv) all sites mapping to high confidence heterozygous or homozygous genomic calls using paired WGS data (*e.g*. BrainVar); v) *de novo* called sites adjacent to read ends and splice sites; vi) *de novo* called sites if coverage was below ten reads, edited read coverage was below three reads and the editing ratio was below 1%; vii) *de novo* sites if called by only one software; viii) supervised sites if coverage was below five reads and the number of edited reads was below three reads. Following, all remaining sites were annotated using ANNOVAR^78^ to gene symbols using RefGene, repeat regions using RepeatMasker v4.1.1, known RNA editing sites using the most recent version of REDIportal^43^ and conservation metrics were gathered using phastCons from the PHAST package.^79^

To classify common, postnatal and prenatal-specific editing sites, samples were subset from each brain region into three groups containing either all samples, prenatal samples or postnatal samples. Next, for each subset, high-quality sites were required to have detection rates in at least 70% and a minimum mean editing level of 5% across all subsetted samples. Further, samples containing more than 20% missing values were removed. All resulting RNA editing data frames (whereby rows are editing sites and columns are samples) contained no more than ~6% missing data on average. For sites classified as ‘common’, any missing values were imputed using predictive mean matching method in the mice R package,^80^ using five multiple imputations and 30 iterations.

#### Computing the Alu editing index (AEI)

The AEI method v1.0^39^ computed the *Alu* editing index (AEI) using an STAR mapped bam file as input. The AEI is computed as the ratio of edited reads (A-to-G mismatches) over the total coverage of adenosines in *Alu* elements and is a robust measure that retains the full *Alu* editing signal, including editing events residing in low-coverage regions with a low false discovery rate. The resulting metric is multiplied by 100 to compute so the index describes the percentage level of editing. For human and macaque RNA-seq samples, predetermined genomic regions were set to all SINE/*Alu* repeats using the *Alu* bed table of the UCSC genome browser defined by RepeatMasker. For mouse, these genomic regions were set to all B1-SINE and B2-SINE elements, where most RNA editing occurs in mouse. Common genetic variation was also discarded for all species using coordinates from UCSC genome browser (hg38 CommonGenomicSNPs150, rheMac8 CommonGenomicSNPs0.01, mm10 CommonGenomicSNPs142). Notably, the AEI has is scalable and comparable across postmortem brain RNA-seq samples generated across unique laboratoaries and library preparation protocols.^59^

#### Cellular deconvolution of bulk brain transcriptome samples

To identify changes in cellular composition in bulk RNA-seq data, non-negative least squares was applied from the MIND R package^92^ and utilized the default Darmanis et al., signature matrix which contained a mixture of six major cell types^93^: mature (adult) neurons, immature (fetal) neurons, astrocytes, oligodendrocytes, oligodendrocyte precursor cells (OPCs) and microglia. NNLS, executed through the est_frac function, was applied to log_2_ count per-million (CPM) transformed data using the *limma* package in R.^94^ Our predictions were focused on these major cell types to reduce noise and to evaluate a distribution of cell type changes that reflect an approximate expected distribution based on prior work.

#### Differential RNA editing analysis and functional annotation

To identify sites with differing editing levels across cortical development, linear modeling via the *limma* R package^94^ was implemented and covaried for the possible influence of sex and ancestry. Given ADAR enzymes are dynamically regulated across development and RNA editing is a process that is highly abundant in neurons, which also increase in frequency through development, additional models were fit to adjusted for *ADAR1*, *ADAR2* and estimated neuronal proportions. All significance values were adjusted for multiple testing using the Benjamini and Hochberg (BH) method to control the false discovery rate (FDR). Sites passing a multiple test corrected p-value < 0.05 were labeled significant.

Genes harboring A-to-I sites with significant increases in editing levels across development were functionally annotated. GO ontology terms relevant to cellular components, molecular factors, biological processes and metabolic pathways were explored using the ToppGene Suite software.^82^ A genomic background was defined as all genes harboring at least one editing site and tested for significance using a one-tailed hyper-geometric distribution with a Bonferroni correction. This is a proportion test that assumes a binomial distribution and independence for probability of any gene belonging to any set. A one-sided test was applied to explicitly test for over-representation of genes harboring editing sites across hundreds of GO categories, without any a priori selection of candidate gene sets. Further, SynGO^83^ analysis was performed under default parameters to test for enrichment of pre- and post-synaptic genes for loci harboring A-to-I sites that either increase or decrease in editing levels across development.

#### Enrichment for disorder-related genes and RNA editing sites

Genes harboring sites that increase in editing levels across development were interrogated for over-representation of neurodevelopmental and neuropsychiatric disorder-related genes. Four tiers of gene sets were collected to examine overlap with co-editing modules: (1) whole exome sequencing (WES)-derived gene sets implicated in risk for autism spectrum disorder (ASD),^95^ schizophrenia^96^ and intellectual disability (ID)^97^; (2) genome-wide association study gene sets (gene(s) nearest candidate risk variants) that implicate risk for ASD,^98^ EA,^99^ schizophrenia^100^ and height^101^ (as a negative control); (3) transcriptome-derived gene sets of RNA editing sites associated with human cortical development,^26^ schizophrenia,^27^ Fragile X Syndrome and ASD.^44^ To compute significance of all intersections, GeneOverlap function in R^102^ was leveraged which uses a Fisher’s exact test (FET) and an estimated odds ratio for all pairwise tests based on a background set of genes detected in the current study. When testing overlap across co-editing modules, all pairwise tests were adjusted for multiple testing using BH procedure to control the FDR.

#### Identification and annotation of RNA hyper-editing sites

To identify hyper-edited reads in the current study, all discarded and unmapped reads following STAR alignment were converted to FASTQ format and used as input for hyper-editing analysis. A well-validated RNA hyper-editing pipeline was applied,^21,45^ consisting of multiple steps: (1) All unmapped reads were removed by phred (<25), the presence of simple repeat structures, high *N* content, or the fraction of a unique nucleotide per read being too great (>60%) or too low (<10%); (2) for resulting reads, all adenosines were transformed into guanosines in both the RNA sequences and genome reference sequence; (3) RNA reads were re-aligned using BWA-aln v0.7.15, all unmapped reads were discarded and subsequently all true adenosines were recovered to identify confident A-to-G edits; (4) filtering of multi-mapped reads by selecting only the location with the largest fraction of A-to-G to all mismatches (providing this fraction was ≥10% higher than in all other locations, otherwise the read was discarded); (5) filtering of hyper-editing clusters to obtain high-quality (Phred ≥ 30) A-to-G mismatches in which the number of A-to-G mismatches was ≥5% of the read length and >60% (80% for read lengths ≤60 bp) of the total number of mismatches; (6) filtering of clusters that were too dense (>90% of read length), A-to-G mismatching contained within the first or last 20% of reads ends and clusters >60% of an individual nucleotide. Once clusters were identified: (7) cluster boundaries were extended by the mean distance between editing sites per cluster and subsequently merged clusters with overlapping coordinates (cluster length is a commonly product of read length); (8) all resulting hyper-editing sites were annotated using ANNOVAR (described above); (9) sites mapping to common genomic variation in dbSNP(v150) and those in gnomAD (maf>0.05) were discarded; (10) sites mapping to paired high-confidence private genomic calls were discarded. This process was reiterated to search for additional hyper-editing substitution types (*e.g*. A-to-C).

To minimize the effect of sequencing batches and differences in library size, a normalized hyper-editing signal was computed. This metric is computed by dividing the total number of hyper-editing sites over the total number of uniquely mapped bases from the initial STAR alignment for each sample and multiplying the result by one million. Picard v2.22.3 collected the number of uniquely mapped bases for each mapped bam file. This enabled us to directly compare normalized hyper-editing levels across independent studies, developmental periods and anatomical regions.

#### Differential analysis of gene-centric RNA hyper-editing

To test for temporal changes in the frequency of hyper-editing sites at the gene level, genes were first filtered according to detection of at least one RNA hyper-editing site in at least 70 DLPFC samples (~40%). This unsupervised measure was used to remove genes with too few hyper-editing sites across all samples. All remaining missing values were treated as zero. Subsequently, the linear modeling framework of *limma*^94^ tested for prenatal and postnatal differences in the frequency of RNA hyper-editing events per gene and adjusted for the possible influence of sex and ancestry. Additional models were fit that covaried for the proportions of mature neurons, *ADAR1* and *ADAR2* expression and gene length. To adjust for gene length, these analyses were performed on a residualized matrix whereby the total number of edits per gene were normalized by the log gene length (geneEdits ~log(gene length)). All significance values were adjusted for multiple testing using the Benjamini and Hochberg (BH) method to control the false discovery rate (FDR). Sites passing a multiple test corrected p-value < 0.05 were labeled significant.

#### miRNA binding predictions

The following approach was applied to estimate differences of miRNA minimum free energy on edited versus un-edited 3′UTRs: (1) For each 3′UTR editing site, two 101bp sequences (50bp +/− the target editing site) were obtained, where the only difference between these sequences was either an unedited (A) or edited (G) site; (2) We used miRANDA^85^ to compute local alignments of 3′UTR sequences against all mature miRNAs obtained from miRbase (https://www.mirbase.org); (3) miRANDA computes the stability of the resulting RNA duplex and minimum free energy (DG kal/mol); (4) This process was re-iterated for high confidence alignments that occur in miRNA seed regions (setting –strict parameters). A Mann-Whitney U test was used to test for significance for differences in minimum free energy.

#### Predicting cryptic splicing

SpliceAI^86^ computed the probability of intronic sites to be splice altering. This method uses a deep neural network to predicts splice junctions from an arbitrary pre-mRNA transcript sequence. All high-confidence intronic A-to-I sites near exon boundaries were included in this analysis. This approach enables prediction of RNA editing events that cause cryptic splicing and generates two outputs for each site: (1) Delta score, which is the probability of the A-to-I site being splice-altering and ranges from 0 to 1. In the primary publication of this work, a detailed characterization is provided for 0.2 (high recall), 0.5 (recommended), and 0.8 (high precision) cutoffs; (2) Delta position, which conveys information about the location where splicing changes relative to the variant position.

#### Intron retention analyses

To compute the percent of intron retention over development, we applied Systematic Investigation of Retained Introns (SIRI).^87^ STAR mapped bam files were provided input together with strandedness (forward) and read length (100bp). To compute the percent of intron inclusion, inclusion was measuring using the PI junction field, defined by inclusion counts divided by the sum of inclusion and skipping junction counts. We selected only introns with a unique intron annotation (U introns) that are not involved in other alternative processing events. Introns subjected to PI measurement were also required have an intron length greater than and equal to 60 and have a sum of EE + EI + IE reads be greater than and equal to 20, as previously described.^87^

#### mRNA stability and RNA secondary structure

The following approach was used to estimate the impact of hyper-editing on RNA stability: (1) For each hyper-edited region, two ~2100bp sequences (1000bp +/− the center of the hyper-edited region), where the only difference between these two sequences was either an unedited (A) or edited (G) site as called from the hyper-editing pipeline; (2) RNAfold computed the stability of the local RNA structure,^88^ according to minimum free energy (DG kal/mol) predictions for each sequence; (3) Using the resulting Dot-Bracket notations, the degree of double-strandedness was computed for each estimated RNA secondary structure by calculating the percentage of all Watson crick ‘paired bases’ relative to all bases across the entire estimated structure; (4) Next each structure was assigned back to an individual gene and split into four tiers based on gene-level results from the differential hyper-editing analysis: Tier 3 (Adj. p < 1.0 × 10^−10^); Tier 2 (Adj. *p* 1.0 × 10^−10^-1.0 × 10^−5^); Tier 1 (1.0 × 10^−5^-0.05); and Tier 0 (Adj. p > 0.05); (5) Finally, a Mann-Whitney U test was used to test for significance for differences in minimum free energy and degree of double-strandedness in Tier 3 relative to the remaining tiers.

#### Identification of RNA editing quantitative trait loci

Cis-edQTLs were identified for all high-quality, common variants within 1 Mb (±) of an editing site using the fastQTL permutation based analysis^89^ using a total of 1,000 permutations. Developmental period, sex, *ADAR1*, *ADAR2*, and the first five principal components of common variant ancestry were used as covariates. This analysis was run on three partitions of the DLPFC dataset: the full sample (*n* = 176, periods 1–12), prenatal samples only (*n* = 112, periods 1–6), and postnatal samples only (*n* = 60, periods 8–12). Separately for the results of each analysis, false discovery rate (FDR) was calculated for all gene-variant pairs using the Benjamini-Hochberg procedure. We then classified all edQTLs with FDR ≤0.05 from at least one analysis into one of five categories defined by the temporal specificity of their edQTL effects (as previously described^32^): (1) Constant edQTLs (consistent effects across development) are defined as edQTLs with FDR ≤0.05 in the complete sample analysis, same direction of effect and unadjusted p ≤ 0.05 in both the prenatal and postnatal analyses; (2) Prenatal-predominant edQTLs (strongest effects during prenatal development) are defined as FDR ≤0.05 in the prenatal analysis, unadjusted p > 0.05 in the postnatal analysis; (3) Postnatal-predominant edQTLs (strongest effects during postnatal development) are defined as FDR ≤0.05 in the postnatal analysis, unadjusted p > 0.05 in the prenatal analysis; (4) Prenatal-trending edQTLs were defined as those that did not fit into earlier categories, but had higher prenatal effects (B_Pre_ > B_Post_); and (5) Postnatal-trending edQTLs were defined as those that did not fit into earlier categories, but had higher postnatal effects (B_Post_ > B_Pre_).

#### Co-localization with UK biobank GWAS summary statistics

GWAS summary statics were leveraged for 109 neurological and mental health-related traits and disorders from the UKBioBank^103^ (ftp://share.sph.umich.edu/UKBB_SAIGE_HRC/) along with GWAS summary statics for attention deficit hyperactivity disorder (ADHD),^104^ schizophrenia,^105^ bipolar disorder,^106^ major depressive disorder.^107^ For each set of summary statistics, genome-wide significant (p < 5.0 × 10^−8^) loci were defined by linkage disequilibrium r2 > 0.6 start and end positions and edQTL sites overlapping those loci were considered for analysis. GWAS and edQTL summary statistics (beta, standard error) for SNPs within each GWAS locus were used as input to coloc2,^90^ and posterior probabilities for five hypotheses (H0, no GWAS or edQTL signal; H1, GWAS signal only; H2, edQTL signal only; H3, GWAS and edQTL signal but not co-localized; H4, co-localized GWAS and edQTL signals) were estimated for each locus. Loci with posterior probability for hypothesis H4 (PPH4) between 0.3 and 0.8 were considered to have moderate co-localization while PPH4 ≥ 0.8 was considered to demonstrate strong Bayesian evidence for co-localization.

## Supplementary Material

1

2

3

4

5

6

7

## Figures and Tables

**KEY RESOURCES TABLE:** 

REAGENT or RESOURCE	SOURCE	IDENTIFIER

Biological samples		

See Table S1 for a complete list of all human samples used in the current study	N/A	N/A
See Table S5 for a complete list of all macaque and mouse samples used in the current study	N/A	N/A

Deposited data		

Raw RNA-seq & WGS data – DLPFC	https://www.synapse.org	syn21557948
Raw RNA-seq data – Cerebrum & Cerebellum	https://www.ebi.ac.uk/arrayexpress/	E-MTAB-6814
Raw RNA-seq data – Cortex and normal aging	https://www.synapse.org	syn7416949
Raw RNA-seq data – hESC differentiation	https://www.ncbi.nlm.nih.gov/geo/	GSE56796
Raw RNA-seq data – hiPSC differentiation	https://www.ncbi.nlm.nih.gov/bioproject/	PRJNA596331
Raw RNA-seq data – Rhesus Macaque	https://www.ncbi.nlm.nih.gov/bioproject/	PRJNA448973
Raw RNA-seq data – Mouse	https://www.ncbi.nlm.nih.gov/sra	SRP055008
Processed RNA editing calls – DLPFC, Cerebrum & Cerebellum	https://www.synapse.org	syn26434508

Software and algorithms		

Code used to process data	This paper	https://doi.org/10.5281/zenodo.7108745
STAR v2.7.3	Dobin etal., 2013^77^	https://github.com/alexdobin/STAR
REDItools v2.0	Flati et al., 2020^40^	https://github.com/BioinfoUNIBA/REDItools2
JACUSA2	Piechotta et al., 2022^41^	https://github.com/dieterich-lab/JACUSA2
SAMtools v1.16	Harvard Medical School, Boston	https://github.com/samtools/samtools
Picard v.2.22.3	Broad Institute, Boston	https://github.com/broadinstitute/picard/
ANNOVAR (Oct 24 2019)	Wang etal., 2010^78^	https://github.com/WGLab/doc-ANNOVAR
Phastcons v.3.15	Hubisz et al., 2011^79^	https://bioconductor.org/packages/release/data/annotation/html/phastCons100way.UCSC.hg38.html
Mice: Multivariate Imputation by Chained Equations v.3.14.0	Buuren etal., 2010^80^	https://cran.r-project.org/web/packages/mice/index.html
RNAEditingIndexer v.1	Roth etal., 2019^39^	https://github.com/a2iEditing/RNAEditingIndexer
bMINDv 0.3.2	Wang etal., 2021^81^	https://github.com/randel/MIND
limma v.3.36.3	N/A	https://bioconductor.org/packages/release/bioc/html/limma.html
ToppGene Suite software	Chen et al., 2009^82^	https://toppgene.cchmc.org/enrichment.jsp
SynGO	Koopmans et al., 2019^83^	https://www.syngoportal.org
GeneOverlap v.1.32.0	Shen et al., 2022^84^	https://bioconductor.org/packages/release/bioc/html/GeneOverlap.html
RNA hyper-editing v.1	Porath et al., 2014^21^	https://github.com/hagitpt/Hyper-editing
miRANDA v.3.3	Betel etal., 2010^85^	https://bioweb.pasteur.fr/packages/pack@miRanda@3.3a
SpliceAI v.1.3.1	Jaganathan et al., 2019^86^	https://github.com/Illumina/SpliceAI
SIRI v.1	Yeom etal., 2021^87^	https://github.com/Xinglab/siri
Viennarna v.2.4.12 (RNAfold)	Hofacker et al., 2003^88^	https://github.com/ViennaRNA/ViennaRNA
fastQTL v.2.184	Ongen et al., 2016^89^	https://github.com/francois-a/fastqtl
Coloc v2	Dobbyn etal., 2018^90^	https://github.com/Stahl-Lab-MSSM/coloc2

## Highlights

- *Alu* editing activity is tightly regulated and increases across neurodevelopment
- Spatiotemporally regulated sites in 3′ UTRs and coding regions offer functional insights
- Hyper-edited RNA is enriched in the aging brain and stabilizes RNA secondary structures
- *cis*-editing quantitative trait loci have unique regulatory effects in development
