# Supplementary material for: Spatiotemporal and genetic regulation of A-to-I editing throughout human brain development
Source: Cell Rep. Author manuscript; Available in PMC 2022 Nov 28. (PMC9704047; doi:10.1016/j.celrep.2022.111585)
Supplement: 1 [file NIHMS1846970-supplement-1.pdf]

Cell Reports, Volume *41*

Supplemental information

Spatiotemporal and genetic regulation of A-to-I editing throughout human brain development

Winston H. Cuddleston, Xuanjia Fan, Laura Sloofman, Lindsay Liang, Enrico Mossotto, Kendall Moore, Sarah Zipkowitz, Minghui Wang, Bin Zhang, Jiebiao Wang, Nenad Sestan, Bernie Devlin, Kathryn Roeder, Stephan J. Sanders, Joseph D. Buxbaum, and Michael S. Breen

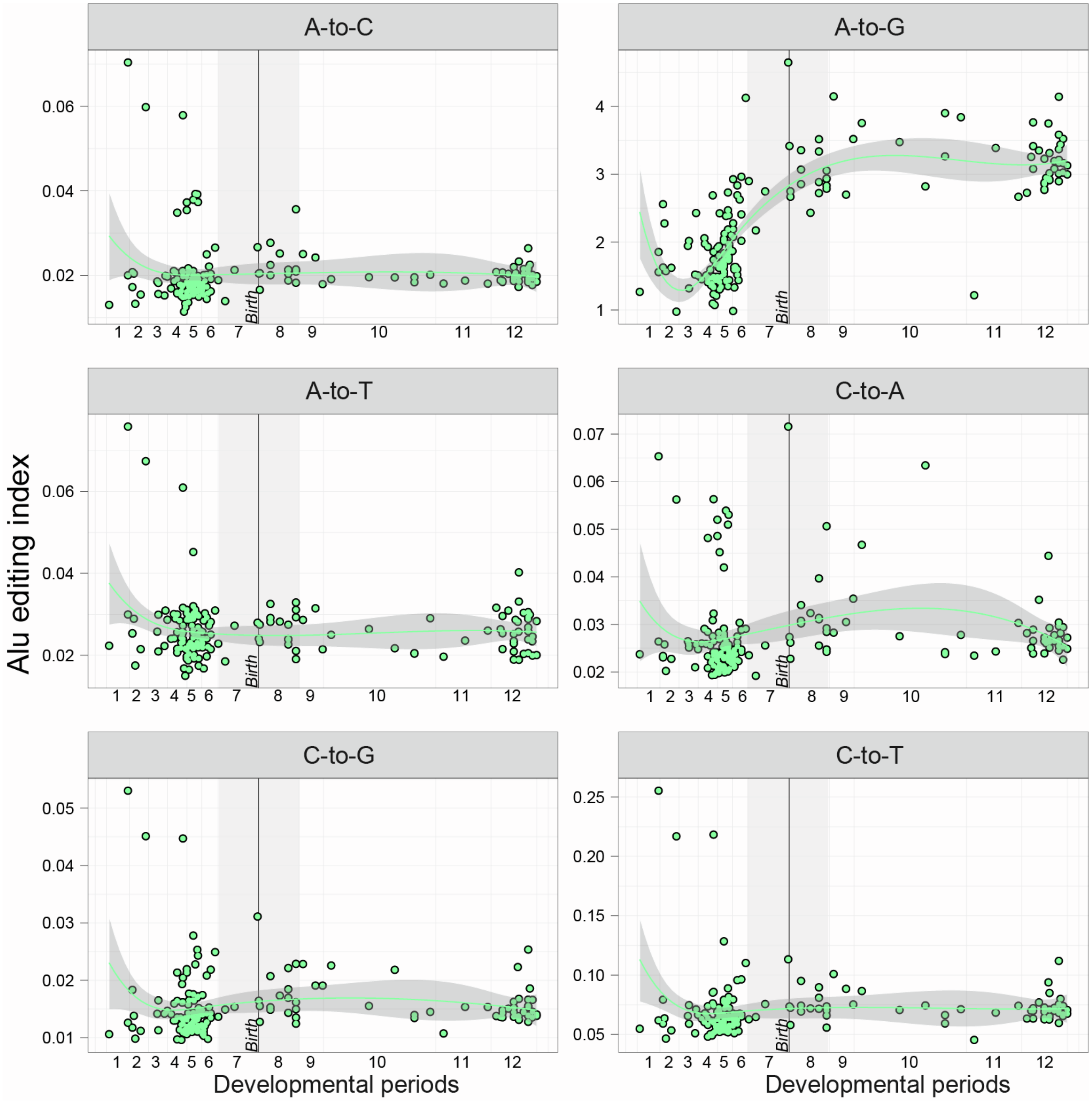

Figure S1. *Alu* editing index by substitution types. DLPFC transcriptome samples (n=176) across prenatal and postnatal development. For each transcriptome sample, we computed the *Alu* editing index (y-axis) defined as ratio of the number of A-to-G mismatches over the total coverage of adenosines in *Alu* elements. The AEI is multiplied by 100 so the index describes the percentage of global editing. We also computed this metric for five additional substitution types across twelve developmental periods (log age, x-axes), which provides an estimate for noise levels. The late fetal transition (epoch 2) is shaded in grey. A loess curve was used to fit the data and standard error is depicted in grey. This supplemental figure is related to Figure 1.

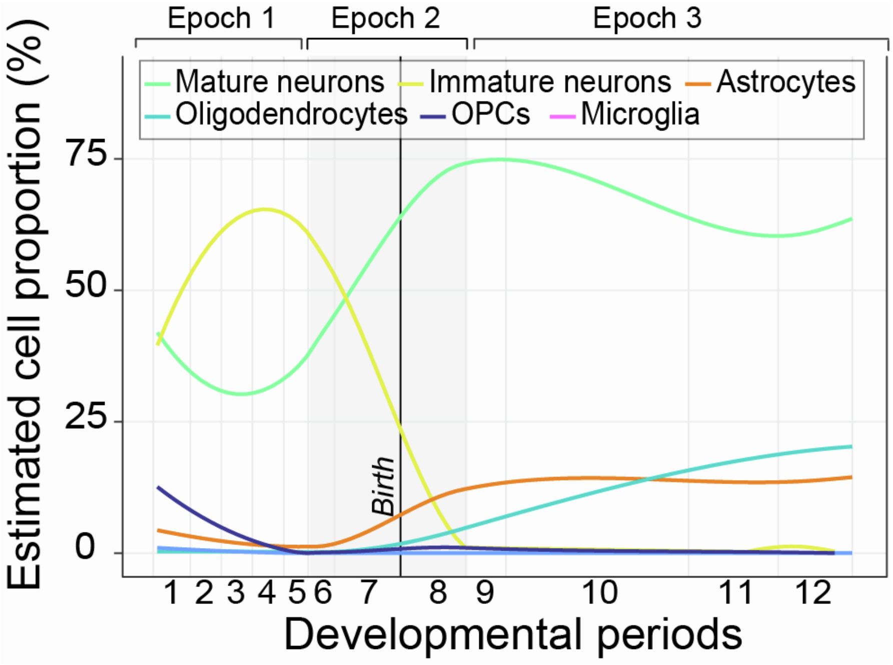

Figure S2. Cellular deconvolution of bulk DLPFC transcriptome samples. Estimated cell type proportions (y-axis) based on cell-specific signatures from Darmanis et al., 2015 were computed for each DLPFC sample and examined across development (log age, x-axis). Periods 1-7 reflect prenatal windows and periods 8-12 reflect postnatal windows. The late fetal transition (epoch 2) is shaded in grey. A loess curve was used to fit the data. This supplemental figure is related to Figure 1.

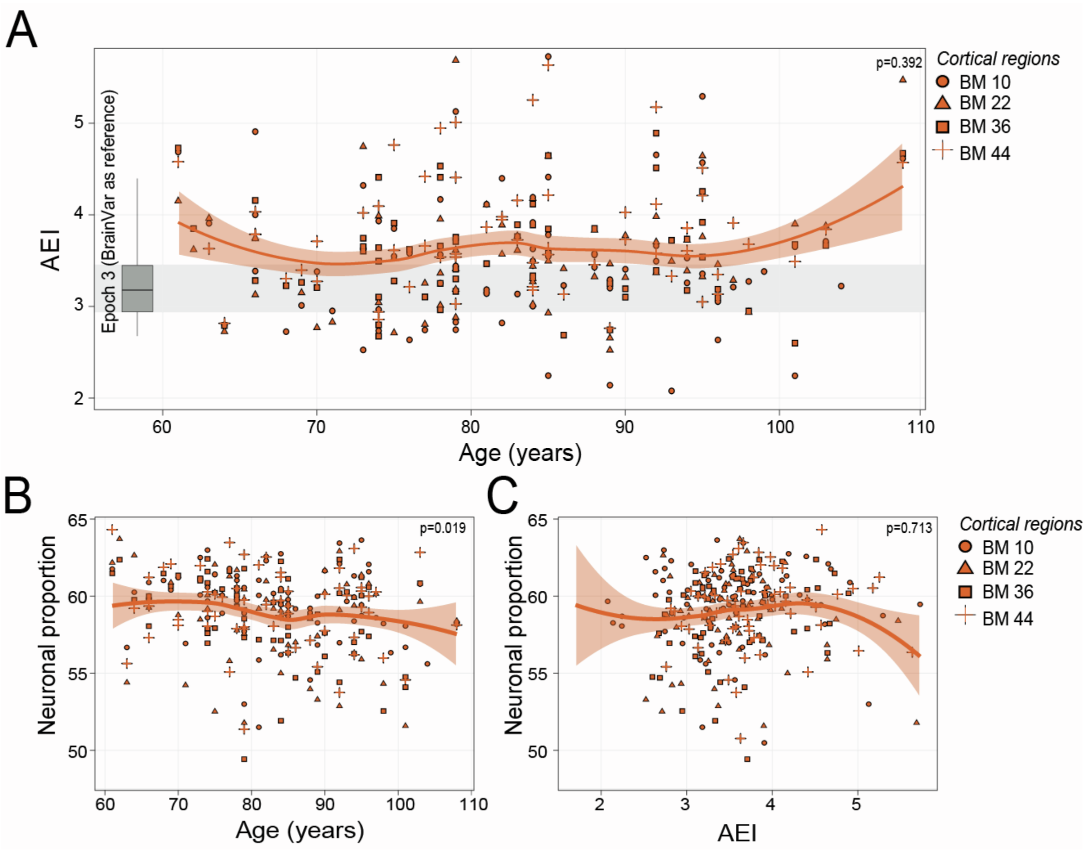

Figure S3. Features of RNA editing during advanced aging. (**A**) The AEI (y-axis) across four cortical areas throughout advanced stages of aging (years; x-axis). Inset boxplot and grey bar indicate the median AEI for the 3rd epoch of the DLPFC (BrainVar cohort) for comparison. Box plot shows the median (horizontal lines), upper and lower quartiles (inner box edges), and 1.5 × the interquartile range (whiskers). There was no AEI effect by cortical region in this dataset. Association between estimated neuronal cell type proportions (%; y-axes) and (**B**) age in years (x-axis) and (**C**) the AEI (x-axis). For each test, a two-sided linear regression computed significance. A loess curve was used to fit the data and standard errors are shown in orange. This supplemental figure is related to Figure 1.

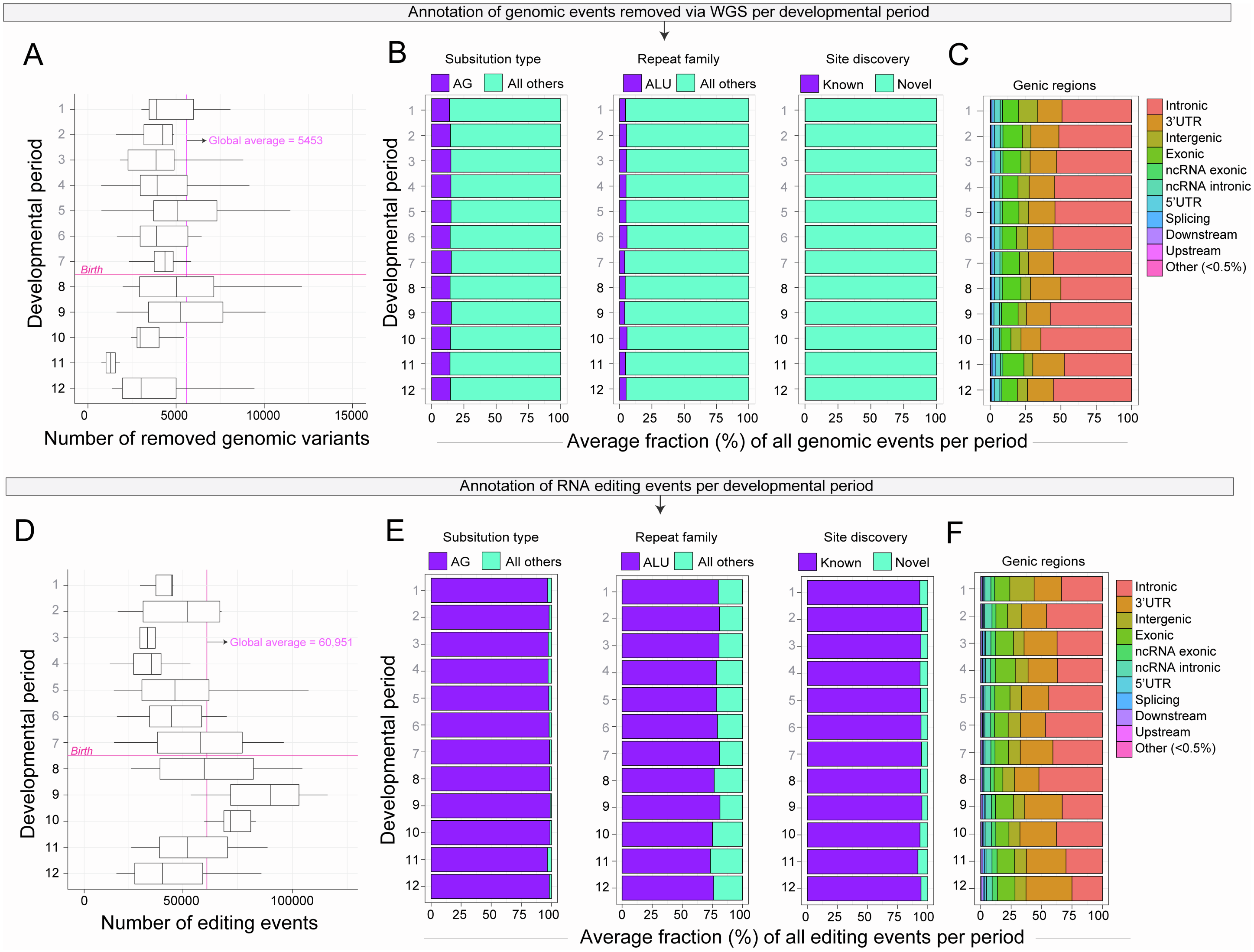

Figure S4. Annotation of sites masked as genomic calls versus true RNA editing sites. Annotation of RNA editing sites in the DLPFC removed following filtering by whole genome sequencing (WGS) data: (**A**) The number of RNA editing sites (x-axis) removed for each developmental period (y-axis). Box plots show the medians (horizontal lines), upper and lower quartiles (inner box edges), and 1.5 × the interquartile range (whiskers). The global average number of RNA editing sites removed by paired WES is marked with a pink line. (**B**) The percentage (%) of all unique genomic sites per period partitioned by i) A-to-G substitutions, ii) sites in *Alu* repeats and iii) known vs. novel RNA editing sites. (**C**) The percentage of all unique genomic editing sites per period according to the corresponding genic regions. Annotation of high-quality RNA editing sites per developmental period: (**D**) The number of RNA editing sites (x-axis) detected for each developmental period (y-axis). Box plots show the medians (horizontal lines), upper and lower quartiles (inner box edges), and 1.5 × the interquartile range (whiskers). The global average number of sites is marked with a pink line. (**E**) The percentage (%) of all unique sites per period partitioned by i) A-to-G substitutions, ii) sites in *Alu* repeats and iii) known vs. novel RNA editing sites. (**F**) The percentage of all unique editing sites per period according to the corresponding genic regions. This supplemental figure is related to Figure 2.

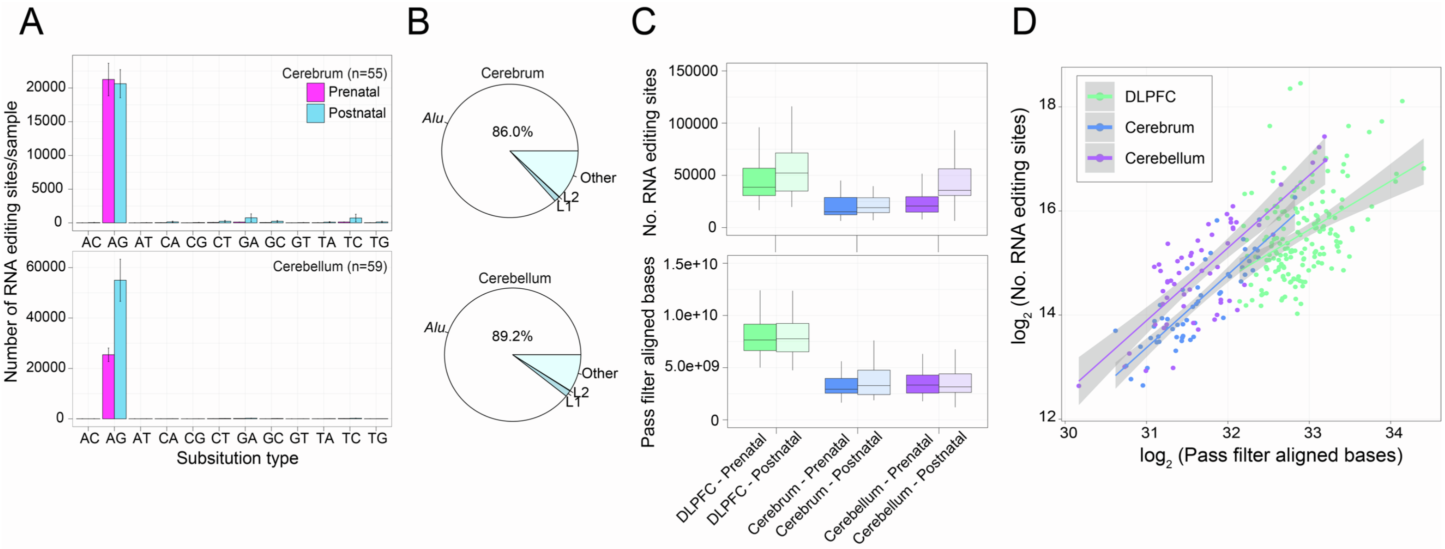

Figure S5. RNA editing site detection, annotation and sequencing depth. (**A**) The mean (and standard error) number of high-quality RNA editing sites detected by modification type for all prenatal and postnatal samples, respectively in the cerebrum (top) and cerebellum (bottom). (**B**) The mean fraction of all high-quality RNA editing sites that map to *Alu* elements, L1, L2 elements, and other repeat elements for the cerebrum (top) and cerebellum (bottom). (**C**) The total number of high-quality RNA editing sites detected (upper) and the number of pass filter high-quality STAR aligned bases (lower; computed using Picard tools) for all DLPFC, cerebrum and cerebellum samples. Box plots show the medians (horizontal lines), upper and lower quartiles (inner box edges), and 1.5 × the interquartile range (whiskers). (**D**) Association between the number of high-quality RNA editing sites (y-axis; logarithmic) and the number of pass filter high-quality aligned bases (x-axis; logarithmic) for each brain region. Linear regression was used to fit the data. Standard errors are depicted in grey. This supplemental figure is related to Figure 2.

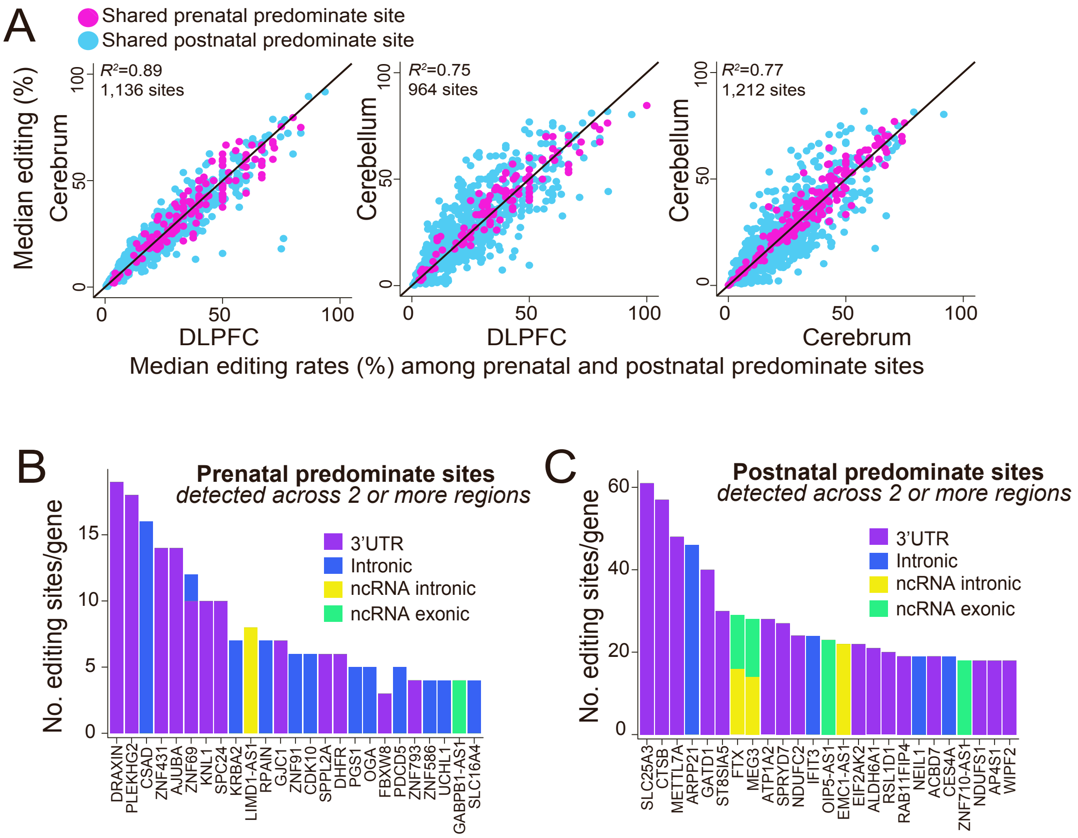

Figure S6. Prenatal and postnatal specific editing sites. (**A**) Pairwise correlations of prenatal and postnatal specific editing sites shared across two or more regions. Concordance was evaluated using the median RNA editing levels computed across all prenatal and postnatal samples, respectively, for each region. For each comparison, the total number of RNA editing sites shared between the two regions is listed. The total number of (**B**) prenatal specific and (**C**) postnatal specific RNA editing sites per gene (y-axes) across the top 25 genes (x-axis). Different genic regions are color coded. Sites were included only if detected across two or more regions. This supplemental figure is related to Figure 2.

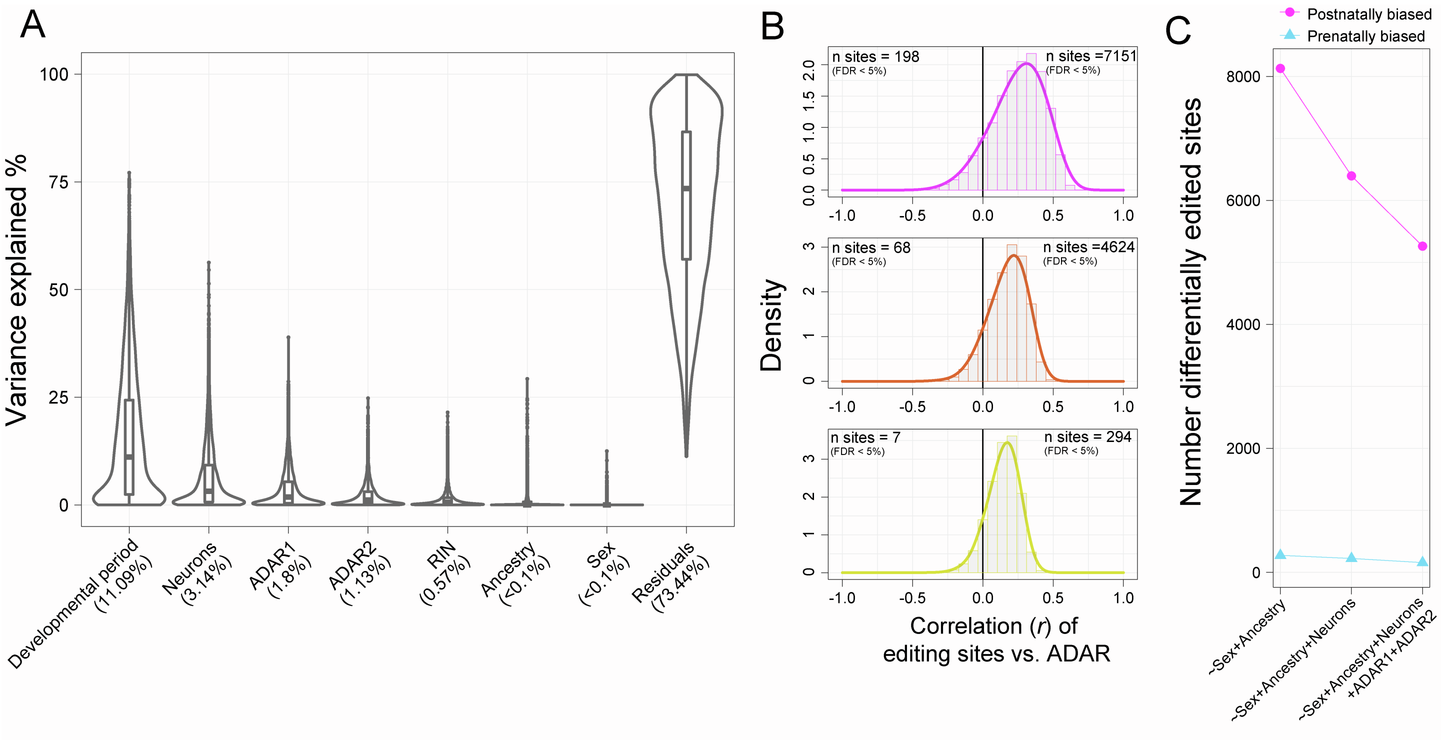

Figure S7. Quantifying variance in RNA editing levels. (**A**) Linear mixed modelling computed the percentage of RNA editing variance explained (%; y-axis) according to seven known factors, which represent potential sources of variability (x-axis). Differences in developmental age, neuronal cell type proportions and *ADAR* expression explains the largest amount of variability. Median levels of explained variation are plotted below. (**B**) Pearson correlation coefficients for *ADAR1*, *ADAR2* and ADAR3 as associated with 10,027 editing sites. The total number of significant correlations passing multiple test correction that are either positively correlated (upper right corner) or negatively correlated (upper left corner) are displayed for each association. (**C**) The number of temporally regulated editing sites (Adj. $P < 0.05$) (y-axis) following a combination of covarying for different factors (x-axis). Differential editing was computed using a moderated t test in the limma R package (*see Methods*). This supplemental figure is related to Figure 3.

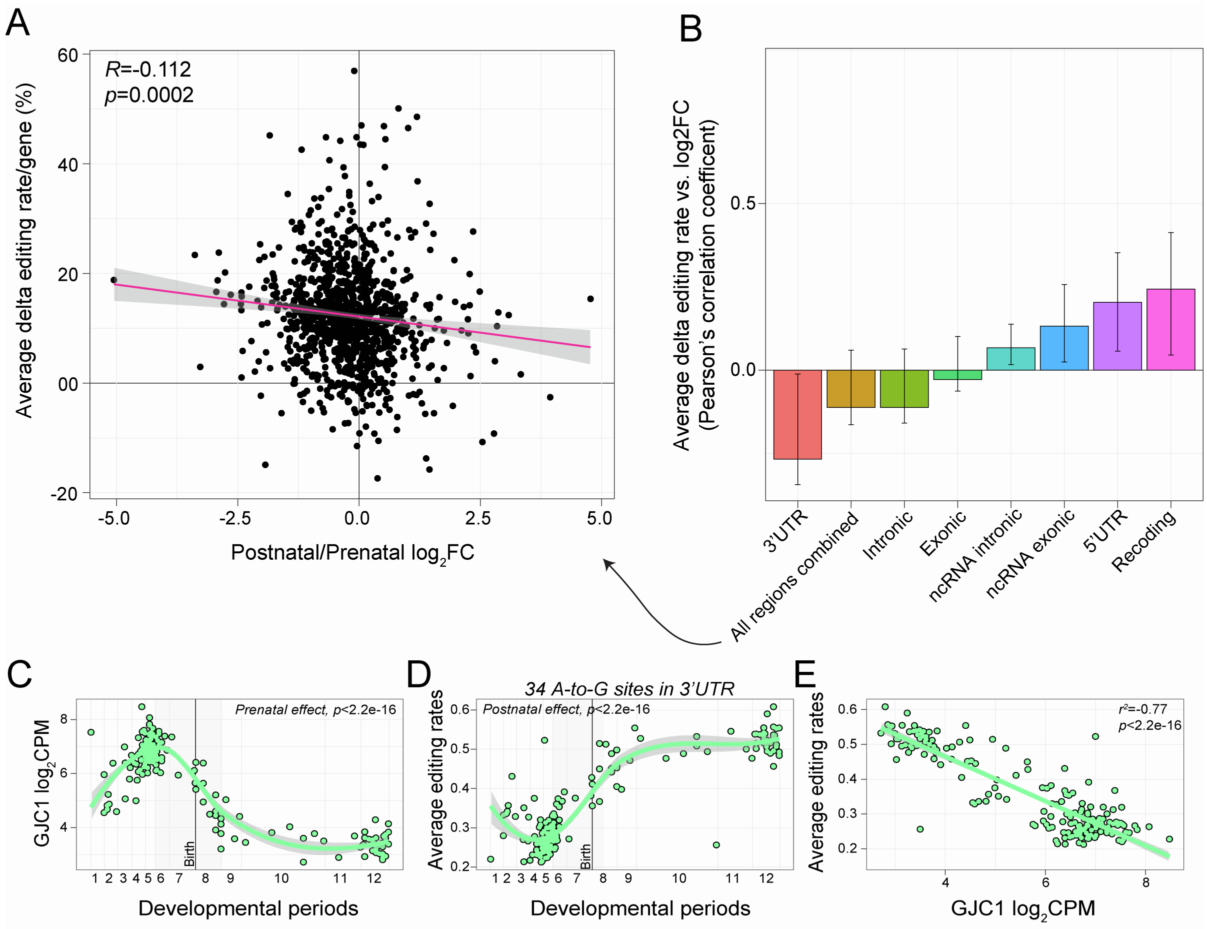

Figure S8. RNA editing levels and gene expression profiles across development. (**A**) Prenatal versus postnatal differences in in RNA editing levels (delta editing rates/gene) were correlated to corresponding gene expression level changes (log fold-change/gene). Delta editing rates per gene were averaged across all delta value for each site per unique gene. Correlation was measuring using a Pearson's correlation coefficient (upper top corner). (B) This analysis was repeated by averaging delta editing levels per site to each unique gene based on the genic location of a site (*i.e.* averaging delta editing levels for sites located only in 3'UTRs/gene). Pearson's correlation coefficient's and 95% confidence intervals are plotted for each genic region. *GJC1* (Gap Junction Protein Gamma 1) is an example of a gene that is (**C**) prenatally biased in expression, but has (**D**) 34 A-to-G editing events in the 3'UTR, that all increase in editing levels over development, and (**E**) the RNA editing levels and gene expression profiles across development are strongly negatively associated (*i.e.* more editing in 3'UTR reflects reduced expression). A loess curve was used to fit the data and standard error is depicted in grey (for panels C and D). A linear regression model was used to fit the data (for panel E). This supplemental figure is related to Figure 3.

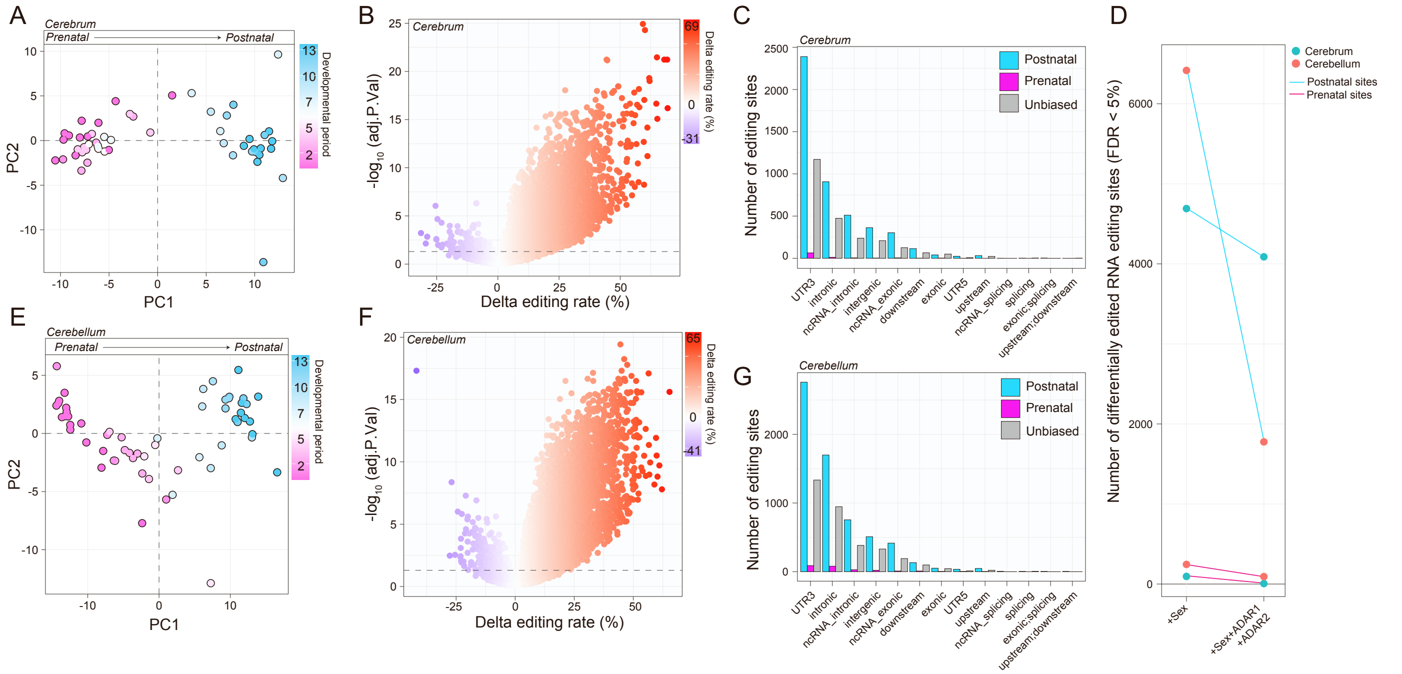

Figure S9. Independent validation of temporal RNA editing profiles in the cerebrum and cerebellum. (A) Principal component analysis of RNA editing sites (n=7,155 sites) across all cerebrum samples stratifies prenatal from postnatal periods. (**B**) Differential RNA editing analysis in the cerebrum compares the strength of significance (-$\log_{10}$ FDR-adjusted P; y-axis) of temporally regulated sites relative to delta editing levels (x-axis). (**C**) Cerebrum sites partitioned according to temporal bias and genic regions. (**D**) The number of temporally regulated editing sites in the cerebrum and cerebellum (Adj. $P < 0.05$) (y-axis) following a combination of covarying for different factors (x-axis). Differential editing was computed using a moderated t test in the limma R package (*see Methods*). (**E**) Principal component analysis of RNA editing sites (n=7,155 sites) across all cerebellum samples stratifies prenatal from postnatal periods. (**F**) Differential RNA editing analysis in the cerebellum compares the strength of significance (-$\log_{10}$ FDR-adjusted P; y-axis) of temporally regulated sites relative to delta editing levels (x-axis). (**G**) Cerebellum sites partitioned according to temporal bias and genic regions. This supplemental figure is related to Figure 3.

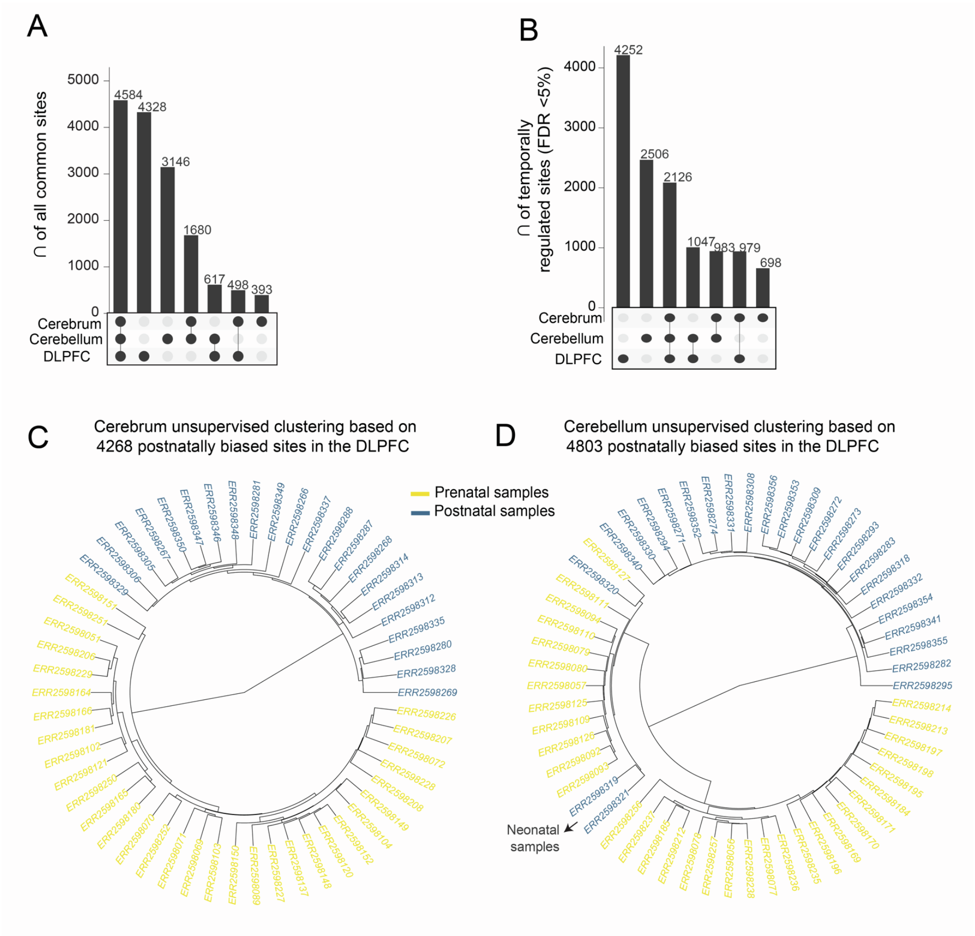

Figure S10. Spatiotemporally regulated editing sites across human brain development. Upset plots depicting (**A**) the convergence of all commonly detected RNA editing sites across the DLPFC, cerebrum and cerebellum data sets and (**B**) the convergence of all significantly temporally regulated sites (FDR < 5%) across each region. (**C**) Unsupervised clustering of cerebrum samples using 4268 overlapping, significant (FDR < 5%) postnatal biased editing sites identified in the DLPFC. (**D**) Unsupervised clustering of cerebellum samples using 4803 overlapping, significant (FDR < 5%) postnatal biased editing sites identified in the DLPFC. This supplemental figure is related to Figure 3.

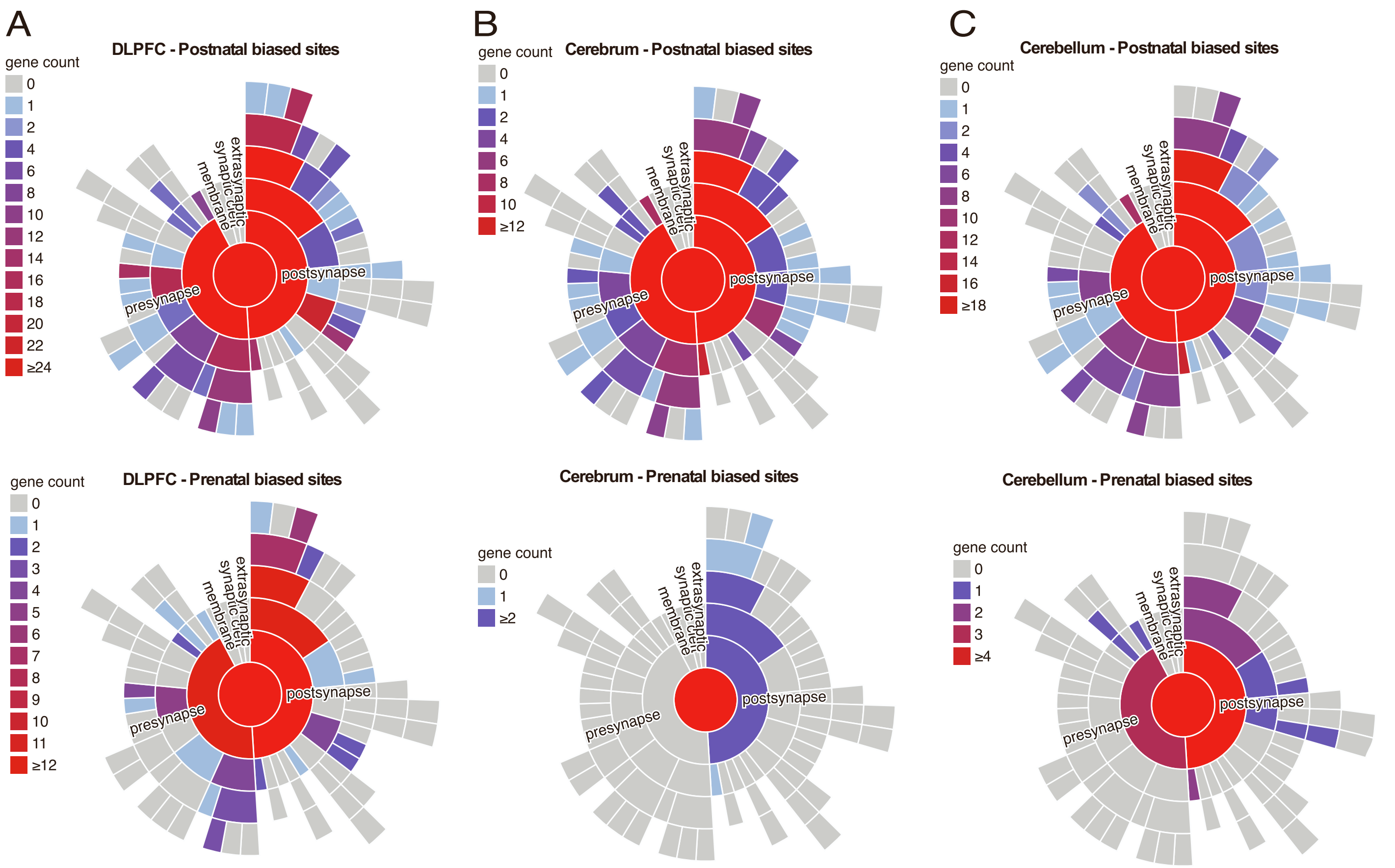

Figure S11. Synaptic gene-set enrichment analysis. SynGo synaptic gene-set enrichment (https://www.syngoportal.org) for genes harboring temporally regulated sites that are either postnatally (top) or prenatally biased in editing levels in the (**A**) DLPFC, (**B**) cerebrum, and (**C**) cerebellum. A general enrichment is observed across all genes harboring postnatally biased sites. This supplemental figure is related to Figure 3.

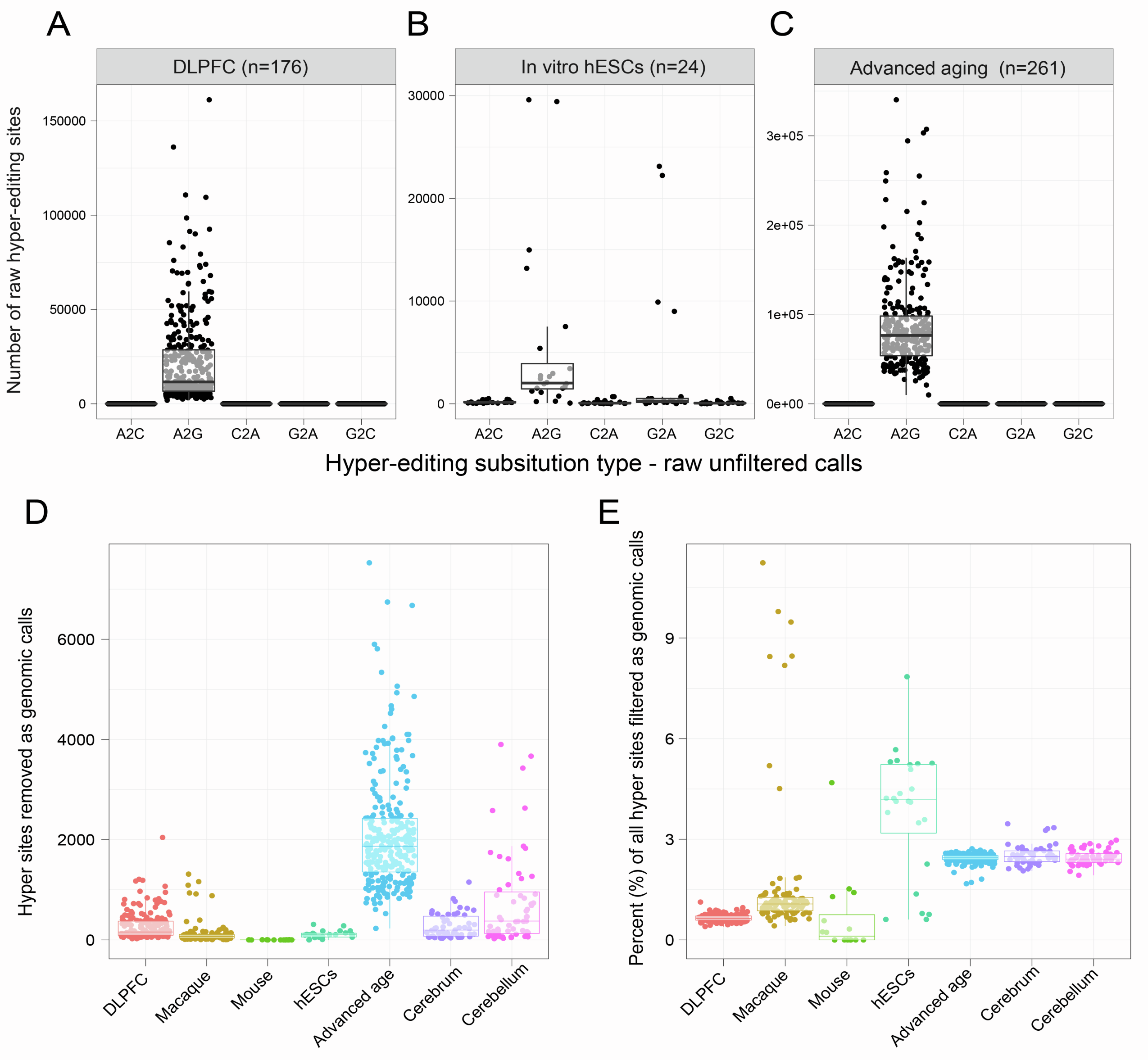

Figure S12. RNA hyper-editing quantification and genomic filtering. Five different hyper-editing substitution types (x-axes) were queried for all samples in the current study. Here we show examples of these calls across all (**A**) DLPFC, (**B**) hESCs and (**C**) cortical samples from advanced aging. A massive A-to-G signal was observed among raw calls prior to further filtering and annotation steps (*see Methods*). **(D)** The total number of A-to-G hyper-editing sites and the (**E**) percentage (%) of all sites removed from across all transcriptome samples (x-axis). Notably, in addition to filtering for common genetic variants in dbSNP, DLPFC samples underwent additional filtering for private genomic variation using paired WGS. Overall, less than ~3% of all hyper-editing sites were removed before proceeding to downstream analyses. This supplemental figure is related to Figure 5 and Figure 6.

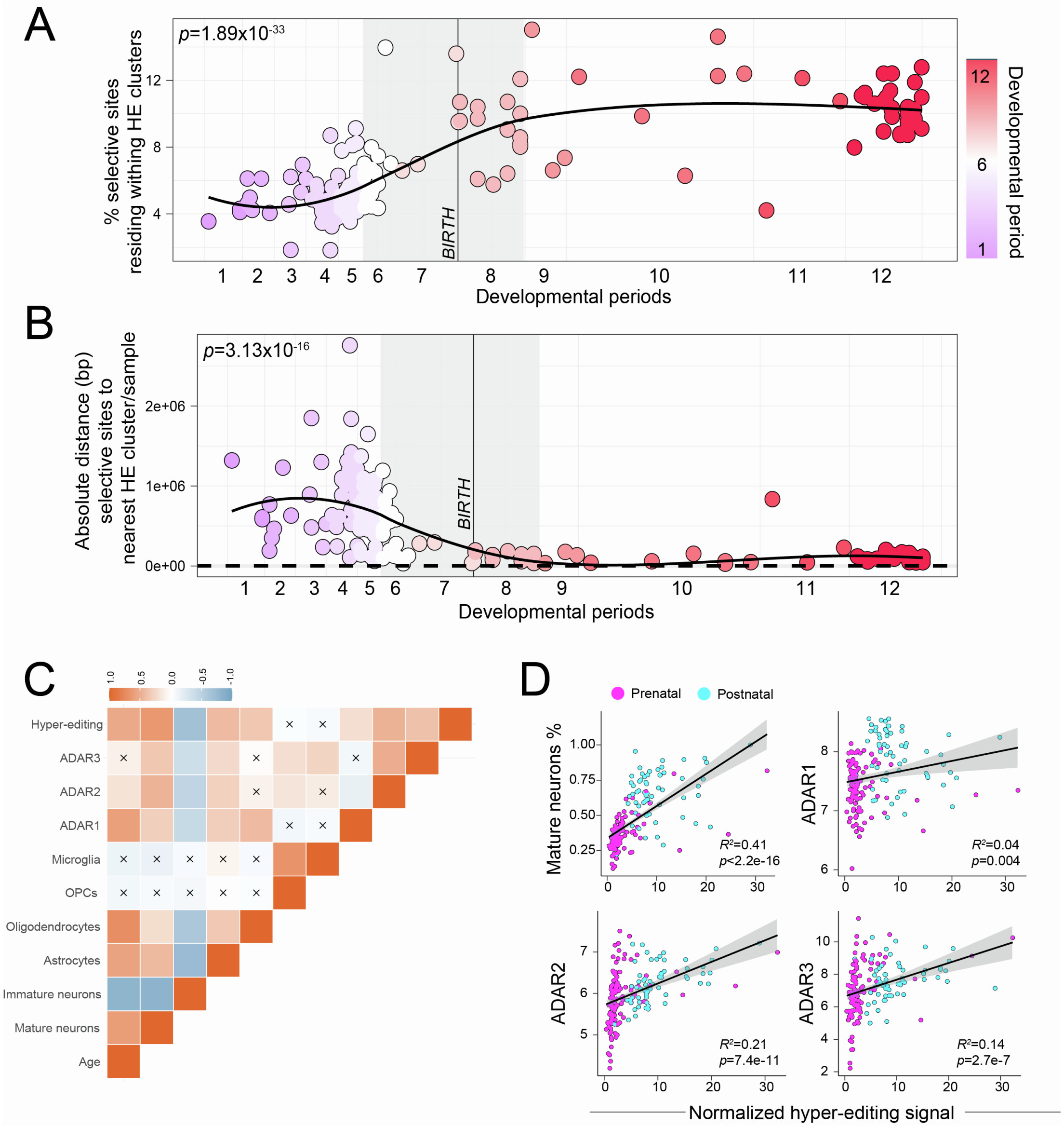

Figure S13. Quantifying RNA hyper-editing variance. (**A**) The percentage of all selective RNA editing sites per sample that reside within paired hyper-editing clusters (y-axis) across DLPFC development (x-axis). (**B**) The average absolute distance (nucleotides) between all selective editing sites per sample to the nearest hyper-edited cluster (y-axis) across DLPFC development (x-axis). A loess curve was used to fit the data and a two-sided linear regression was used to test for significance between all prenatal and postnatal developmental samples. (**C**) Pairwise Pearson's correlations computed associations between the number of filtered high-confidence A-to-G hyper-editing sites with ADAR expression, estimated brain cell type proportions and chronological age. (**D**) Pairwise regression analyses between the normalized A-to-G hyper-editing signal and the estimated proportion of mature neurons, *ADAR1*, *ADAR2* and *ADAR3*. This supplemental figure is related to Figure 5.

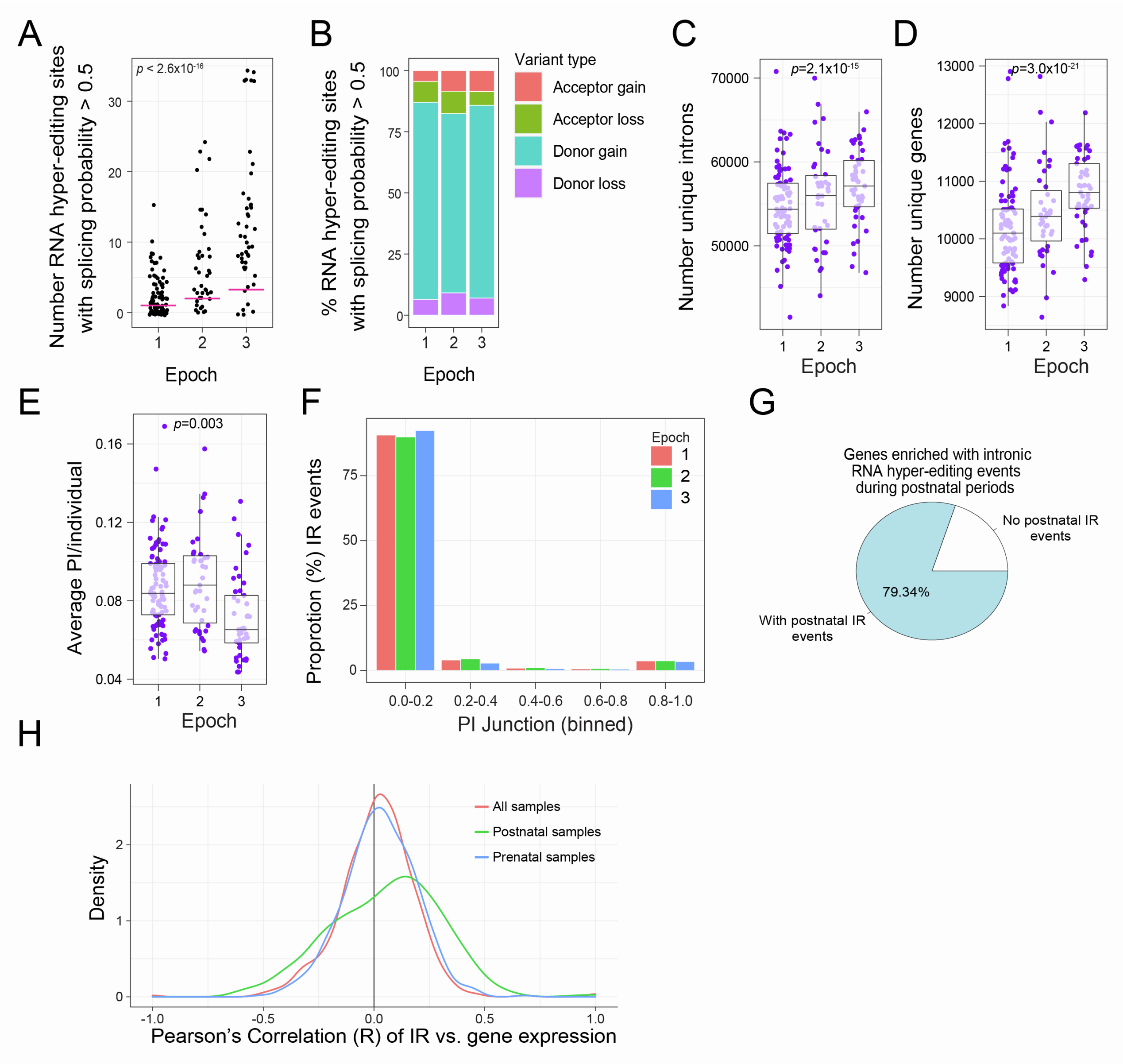

Figure S14. SpliceAI and intron retention predictions. (A) The total number of splice-altering intronic RNA hyper-editing events (y-axis) per donor according to developmental epoch (x-axis). We used a cut-off of ≥ 0.5 as a probability to interpret whether an editing site is splice altering. A linear model tested the association between epoch and number of sites with probability ≥0.5. (**B**) The percentage of all sites with probability ≥0.5 according to their variant type, whereby the majority of splice altering variants were predicted to be donor gain events. The total number of (**C**) unique introns and (**D**) unique corresponding genes with detectable IR. We specifically focused on introns do not partly overlap with exons or with other introns. (**E**) Percent of introns (PI) was averaged across IR events per donor and is defined by inclusion counts divided by the sum of inclusion and skipping junction counts. Analysis of variance was used to test for significance across all three developmental epochs. (**F**) Frequency of PI metrics binned by epoch. (**G**) The fraction of genes enriched with intronic RNA hyper-editing sites during postnatal periods that were also associated with elevated IR. (**H**) Pearson's correlation coefficient of PI metric vs paired gene expression for 583 postnatally hyper-edited genes with measurable IR events. We used the top PI value per gene to compute association with gene expression ($\log_2$CPM). This supplemental figure is related to Figure 5.

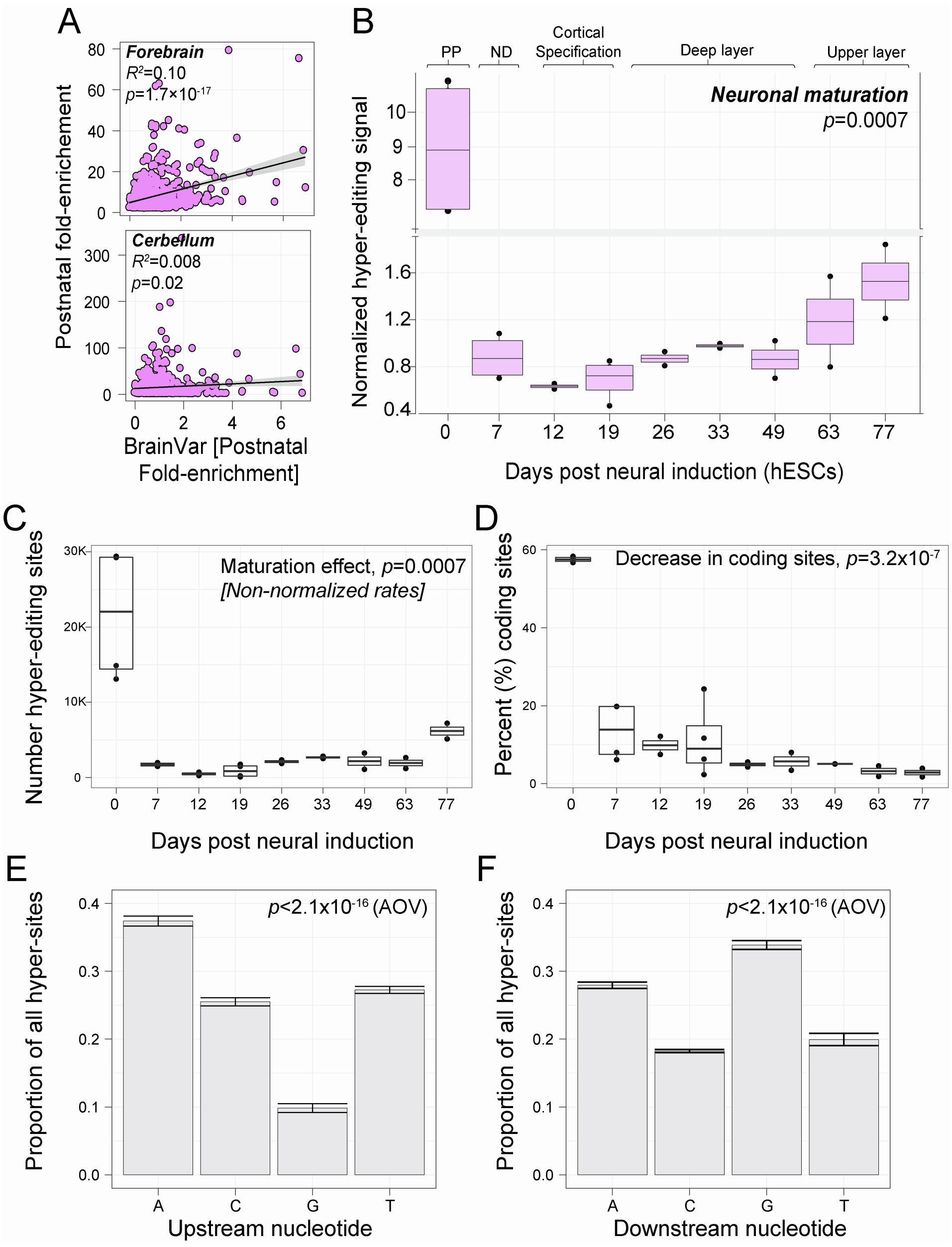

Figure S15. RNA hyper-editing validation per gene and across early corticogenesis. (A) Validation of the postnatal enrichment of RNA hyper-editing signal per gene in the cerebrum (top) and cerebellum (bottom). Pearson's correlation coefficient measured concordance between postnatal fold-enrichment of hyper-editing sites per gene across independent brain regions and datasets. (**B**) The normalized hyper-editing signal through early corticogenesis in hESCs. (**C**) The total number of high-quality A-to-G hyper-editing sites (y-axis) across days post neuronal induction (x-axis). A linear regression tested the number of sites across 77 days in culture. Note that day 0 indicates pluripotency and was dropped from this analysis as it was an extreme outlier in A-to-G hyper editing signal. (**D**) The majority of A-to-G hyper-editing signal during pluripotency occurred in coding regions, which are commonly rare events. Local sequence motifs for all high-confidence A-to-G hyper editing sites indicate a (**E**) depletion of guanosine 1bp upstream and (**F**) an enrichment of guanosine 1bp downstream the target adenosine. Analysis of variance was used to test for significance. This supplemental figure is related to Figure 5.

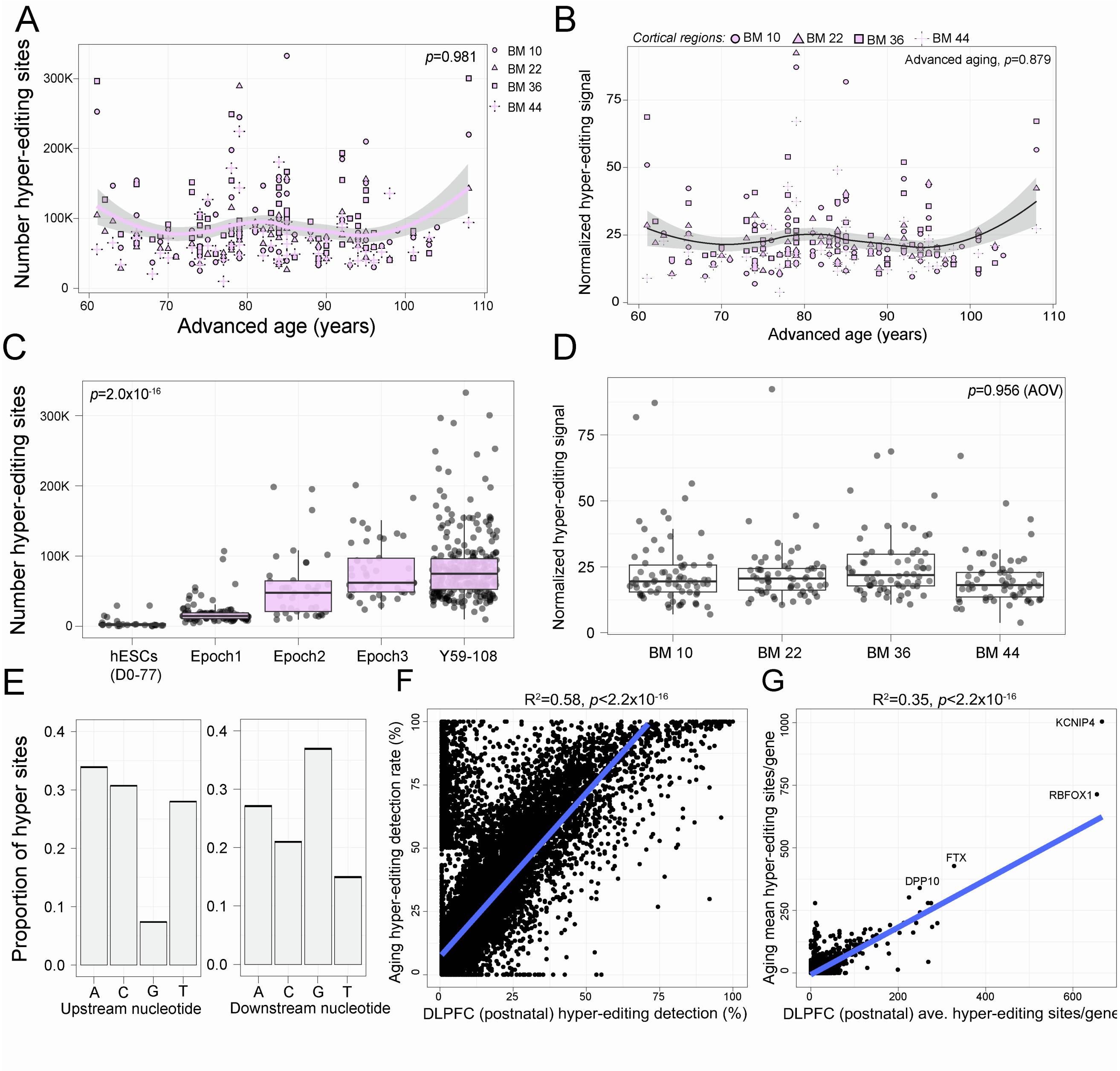

Figure S16. RNA hyper-editing in during advanced age. (**A**) The total number of high-quality A-to-G hyper-editing sites (y-axis) across advanced age (x-axis). (**B**) The normalized RNA hyper-editing rate across advanced age. A linear regression tested the number of sites across 58 years to 108 years of age. (**C**) The total number of high-quality A-to-G hyper-editing sites (y-axis) across all samples, including hESCs (n=24), epoch 1, epoch 2 and epoch 3 (BrainVar), and all advanced age samples (x-axis). A linear regression was used to test for significance. (**D**) Normalized hyper-editing rates do not vary across the four cortical areas. (**E**) Local sequence motifs for all high-confidence A-to-G hyper editing sites indicate a depletion of guanosine 1bp upstream and an enrichment of guanosine 1bp downstream the target adenosine. **(F)** Validation of genes enriched for postnatal hyper-edited. Detection rates (%) of hyper-editing sites per gene in cortex of advanced aging (y-axis) versus postnatal DLPFC samples (periods 8-12) (x-axis). Overall, the frequency of gene-specific hyper-editing was consistent across the two independent cohorts (R^2=0.58). (**G**) The mean number of hyper-editing sites per gene across all DLPFC samples (x-axis) and all cortical samples of advanced ages (y-axis). This supplemental figure is related to Figure 5.
